# A Hydrogen‐Releasing Nanozyme Engineers a Mitochondrial ROS Amplifier for Self‐Sustaining Catalytic Immunotherapy

**DOI:** 10.1002/advs.202524313

**Published:** 2026-04-10

**Authors:** Mingfan Shi, Jingrui Cao, Tong Wu, Guang Yang, YaWen Yang, Shixin Zhang, Wenwen Su, Hongyu Chu, Yangyang Zhao, Shan Jiang, Qiong Wu, Dongxu Jiao, Fangfang Chen

**Affiliations:** ^1^ Key Laboratory of Pathobiology Nanomedicine and Translational Research Center Ministry of Education China‐Japan Union Hospital of Jilin University Changchun Jilin China; ^2^ Huzhou Central Hospital The Fifth School of Clinical Medicine of Zhejiang Chinese Medical University Huzhou Zhejiang Province China; ^3^ The First Research Laboratory Changchun Institute of Biological Products Co., Ltd. Changchun Jilin China; ^4^ JILIN Cancer Hospital Changchun Jilin China; ^5^ Department of Gastrointestinal Colorectal and Anal Surgery China‐Japan Union Hospital of Jilin University Changchun Jilin China; ^6^ College of Chemistry Chemical Engineering and Resource Utilization Northeast Forestry University Harbin China

**Keywords:** hydrogen‐releasing nanozyme, mitochondrial reprogramming, prolonged oxidative stress, Pd‐based alloy nanohydrides, tumor catalytic therapy

## Abstract

The efficacy of nanocatalytic therapy is constrained by the limited availability of endogenous hydrogen peroxide (H_2_O_2_) as a reaction substrate, finite catalytic activity of nanozymes and rapid scavenging by intracellular antioxidants, hindering their accumulation at target sites to therapeutic concentrations. To address the core bottleneck, we developed a hydrogen‐doped rhodium‐palladium alloy (RhPd‑H) nanozyme that integrates enhanced peroxidase (POD)‐mimetic catalytic activity with thermally triggered hydrogen gas (H_2_) release. Under near‐infrared (NIR) irradiation, the RhPd‐H performs POD activity to efficiently produce exogenous hydroxyl radicals (·OH), inducing initial oxidative stress. Concurrently, the released H_2_ flux could reduce the level of reactive oxygen species (ROS) within mitochondria, thereby mitigating oxidative damage and reprogramming mitochondria into endogenous ROS generator that continuously leak superoxide anion (·O_2_^−^). This dual‐path ROS generation mechanism sustains prolonged intracellular ROS burst to efficiently kill tumor cells. Further, in vivo evaluations demonstrated that RhPd‐H nanoenzyme exhibited long‐term tumor retention, significant suppression of tumor growth and activation of antitumor immunity. By differentially regulating ROS across space and time, RhPd‑H nanozyme establishes a persistent and overwhelming oxidative stress that effectively disrupts redox homeostasis. Our work advances beyond conventional catalytic therapy, proposing a new concept of metabolically amplified nanozyme therapy.

## Graphical abstract

We developed a RhPd‐H nanozyme that integrates enhanced POD‐mimetic catalytic activity with thermally triggered H_2_ release. H_2_ shields and reprograms mitochondria to continuously leak endogenous ·O_2_^−^. This dual‐path ROS spatially and temporally disrupts redox homeostasis, inducing persistent oxidative stress for tumor suppression and antitumor immunity, establishing metabolically amplified nanozyme therapy.

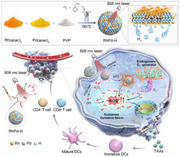

## 1 Introduction

Nanocatalytic therapy represents a pivotal research direction in the field of tumor precision medicine [1−4]. It employs nanomaterials to mimic enzymatic catalytic reactions, enabling the in situ generation of cytotoxic reactive oxygen species (ROS) within the tumor microenvironment (TME), thereby achieving selective killing of cancer cells. Compared to conventional treatments, this strategy is activated by specific TME conditions (e.g., mild acidity, elevated levels of hydrogen peroxide (H_2_O_2_) relative to normal tissues), offering notable advantages including high specificity, low systemic toxicity and reduced risk of drug resistance [5−9]. However, the clinical translation of this approach is hindered by fundamental challenges including limited tumor catalytic substrates and highly active antioxidant defense systems. As a result, ROS generated by a single catalytic process is too weak and short‐lived to cause large‐scale tumor cell death. Furthermore, the heterogeneity and immunosuppressive nature of the TME compromise the thoroughness and systemic efficacy of catalytic treatment [10−14]. Notably, mitochondria as central hubs of cellular energy metabolism and major sources of ROS play a critical role in determining the outcome of catalytic therapy. In conventional approaches, intense and transient exogenous ROS bursts often lead to rapid collapse of mitochondrial membrane potential, dysfunction of the electron transport chain, accompanied by ATP depletion and swift initiation of apoptosis [15−18]. Although such acute damage can induce cell death in a subset of cells, it also abolishes mitochondrial capacity for endogenous ROS generation, limiting therapeutic robustness and persistence of the therapeutic outcome. Therefore, shifting the strategic focus from destroying mitochondria to reprogram their function into endogenous ROS generators that sustain a chronic oxidative stress state would be a promising avenue for improving nanocatalytic therapy.

Hydrogen gas (H_2_) therapy has attracted growing interest in recent years. Hydrogen offers high biosafety, excellent membrane permeability and selective antioxidant properties [19−23]. It can easily penetrate into subcellular structures such as mitochondria. More importantly, hydrogen can mitigate oxidative stress‐induced mitochondrial damage, help maintain relative membrane stability, delay the collapse of the electron transport chain, and regulate mitochondrial metabolism and energy balance. By reprogramming mitochondrial function from “acute collapse” to a state of “sub‐lethal failure”, hydrogen not only protects normal tissues but also induces sustained ROS leakage [24, 25], thereby significantly prolonging and amplifying endogenous ROS production in tumor therapy. The combination of hydrogen therapy and nanocatalysis could establish a self‐enhancing treatment dynamic. Catalytic reactions persistently generate tumor‐attacking ROS, complemented by hydrogen's ability to modulate the ROS landscape and mitochondrial function through selective antioxidant activity. This synergy ensures a more thorough and sustainable therapeutic outcome. Currently, various hydrogen‐generating nanomaterials have been reported for tumor therapy, yet existing systems almost exhibit notable limitations. Although metal hydrides (e.g., CaH_2_, MgH_2_) [26] possess high hydrogen storage capacity, their chemical stability is poor, which often leads to nonspecific hydrogen leakage during physiological circulation. Inorganic non‑metallic carrier materials (e.g., PDA‑based systems) [27] show improved stability and hydrogen loading capacity, but their hydrogen release typically relies on endogenous or external catalysis, which suffers from limited tissue penetration depth and insufficient control precision. While classic metallic hydrogen storage materials like palladium hydride (PdH) [28] are valued for therapeutic potential, achieving a combination of facile synthesis, high hydrogen storage capacity, robust stability and controlled on‐demand release within the same Pd‐H‐based system remains a significant challenge. Additionally, water‐splitting semiconductor materials (e.g., CdS, TiO_2_) [29] commonly face challenges such as severe photocorrosion, poor biocompatibility and low hydrogen production efficiency. Notably, our newly developed hydrogen‐doped rhodium‐palladium alloy (RhPd‐H) demonstrates significant advantages in addressing these limitations: (i) it exhibits superior hydrogen storage stability with no detectable release under ambient conditions, overcoming the instability issues of conventional metal hydrides; (ii) its unique near‐infrared (NIR) light‐responsive property enables spatiotemporally controlled on‐demand hydrogen release, providing precision far beyond non‐metallic carrier systems; and (iii) unlike other Pd‐H‐based nanozymes that typically possess catalase (CAT)‐like and peroxidase (POD)‐like activities or function as broad‐spectrum antioxidants [23, 30, 31], RhPd‐H achieves highly selective single POD activity through Rh incorporation. This eliminates undesirable H_2_O_2_ consumption and enabling efficient conversion of endogenous H_2_O_2_ into toxic hydroxyl radical (·OH), establishing a clean and efficient pro‐oxidant therapeutic mechanism.

**SCHEME 1:**
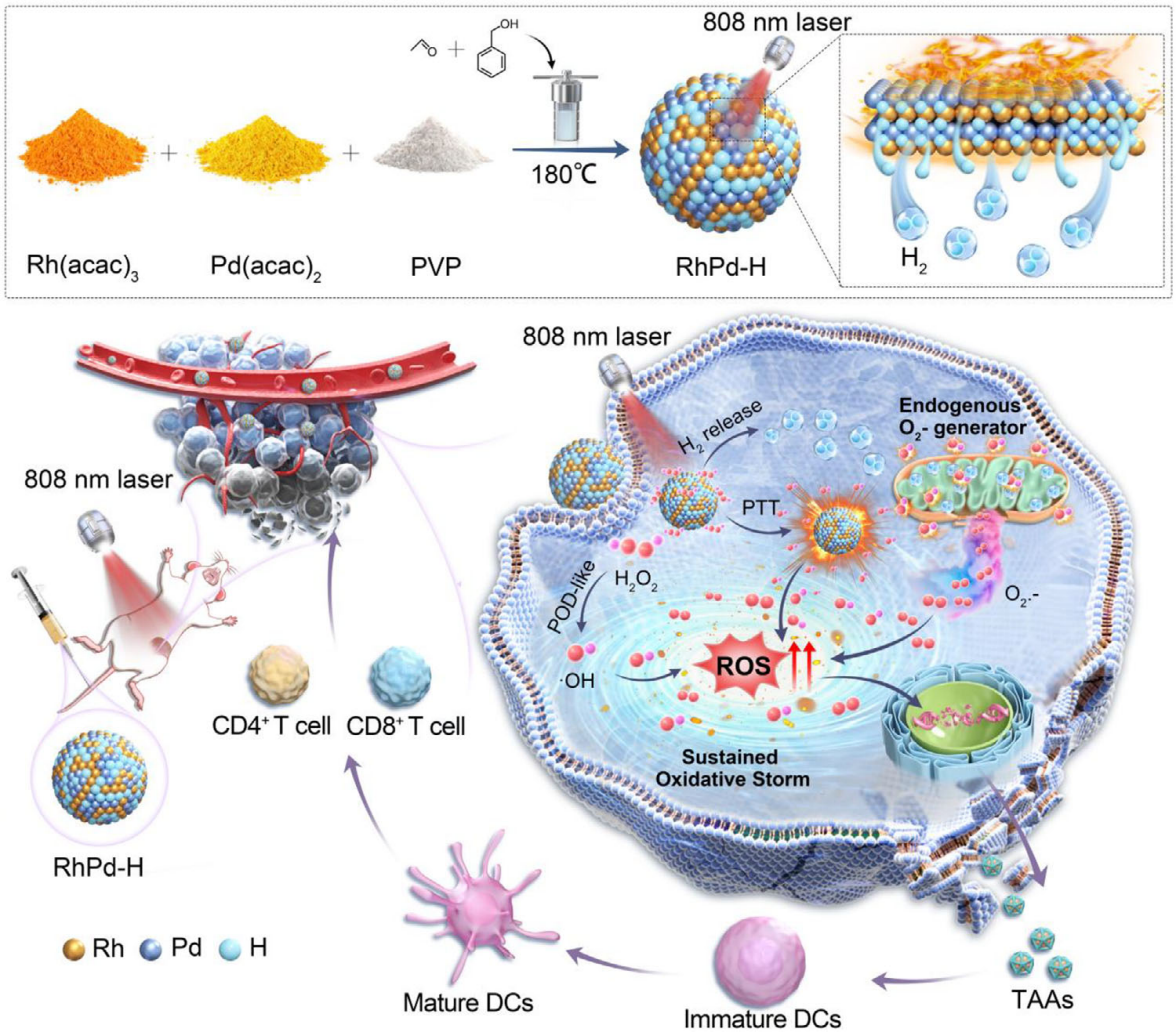
Programmable cascade for self‐amplifying oxidative storm and anti‐tumor immunity orchestrated by RhPd‐H nanozyme. NIR irradiation activates the RhPd‐H nanozyme to concurrently exert photothermal action and POD‐mimetic catalysis, instigating primary oxidative stress. The thermal‐triggered H_2_ gas flux targets mitochondria and reprograms this organelle into a durable endogenous ROS amplifier that drives a cascade‐like and self‐sustaining oxidative storm. This relentless oxidant stress induces robust ICD, facilitating the release of tumor‐associated antigens (TAAs) and DAMPs to activate DCs and recruit cytotoxic T lymphocytes, thereby transforming localized nano‐catalytic treatment into a systemic anti‐tumor immune response.

Accordingly, RhPd‐H enables the integration of hydrogen, photothermal and nanocatalytic therapy into a single platform. Under a single stimulus of NIR light, the localized heat from photothermal therapy (PTT) enhances the catalytic reaction rate and triggers rapid hydrogen release. Meanwhile, the catalytic reaction consumes H_2_O_2_ to produce ·OH, causing an initial oxidative burst. The released H_2_ gas diffuses into mitochondria and helps to scavenge ROS, as demonstrated in several studies where hydrogen‐generating nanomaterials effectively reduced intracellular ROS levels in tumor cells [27, 32]. Notably, this scavenging effect is transient. Following initial ROS reduction, prolonged hydrogen exposure can reprogram mitochondrial function, leading to sustained endogenous ROS generation [33]. This biphasic modulation, characterized by initial protection followed by functional reprogramming, partially preserves mitochondrial structure while inducing electron transport chain inefficiency, creating a “leaky” state that sustains oxidative stress over time. Consequently, mitochondria are reprogrammed into sustained endogenous ROS “secondary amplifiers”. These amplify internally generated ROS in a positive feedback loop with externally supplied ROS from catalytic process, ultimately producing a potent and prolonged ROS burst that efficiently induces cellular apoptosis (Scheme 1). The proposed system exemplifies a spatiotemporally synchronized “catalytic‐thermal‐hydrogen” triple synergistic strategy, which significantly enhances tumor cell killing and initiates a prominent antitumor immune cycle through immunogenic cell death (ICD), dendritic cells (DCs) maturation and T‐cell activation. This work identifies the precise regulation of mitochondrial function by hydrogen as a novel mechanism to potentiate nanocatalytic immunotherapy, representing a paradigm shift in multi‐modal catalytic therapy.

## 2 Results and Discussion

### 2.1 Characterization of RhPd‐H Nanoparticles

**FIGURE 1:**
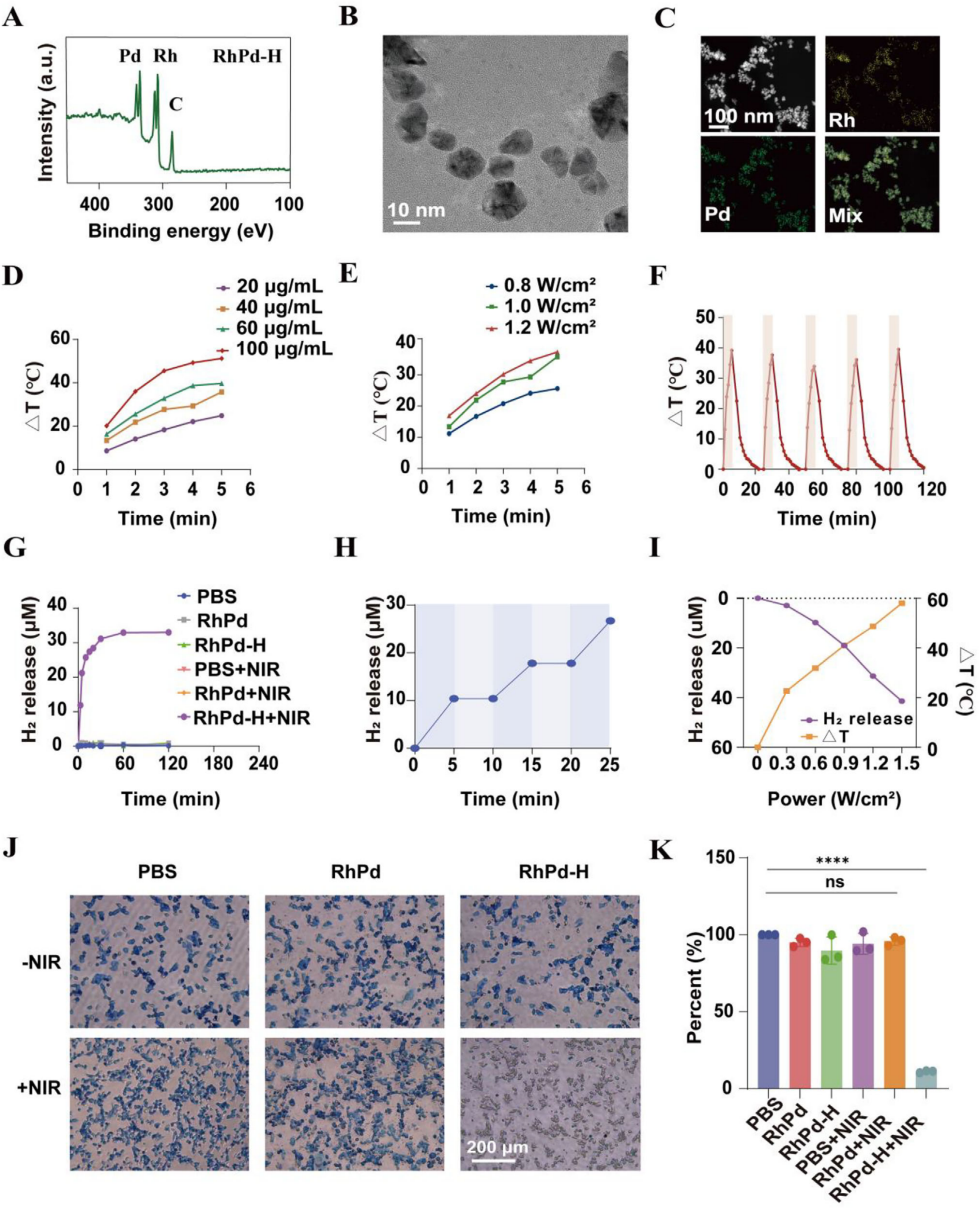
Characterization and performance test of RhPd‐H nanoenzyme. (A) XPS, (B) TEM and (C) HADDF‐STEM and EDX elemental mapping images of the RhPd‐H NPs. (D) Concentration‐dependent temperature elevation of RhPd‐H in PBS under 808 nm laser irradiation (1 W/cm^2^). (E) Heating profiles of RhPd‐H in PBS under 808 nm laser irradiation at varied power densities. (F) Photothermal cycling test of RhPd‐H. (G) Hydrogen release behavior under different treatment conditions. (H) Kinetics and reversibility of the H_2_ release process from RhPd‐H in response to laser switching. (I) Hydrogen release and temperature change curves of RhPd‐H under different powers. (J) Visualization of H_2_ production in 4T1 cells via the methylene blue (MB) probe assay. (K) Quantified intracellular H_2_ levels from the MB assay (*n* = 3). Statistical significance was determined by a two‐tailed Student's t‐test: ^*^ *p* < 0.05, ^**^ *p* < 0.01, ^***^ *p* < 0.001, ^****^ *p* < 0.0001.

A systematic characterization of the synthesized RhPd‐H nanostructure was conducted to confirm their fundamental structures, elemental composition, stability, and biocompatibility. X‐ray photoelectron spectra (XPS) analysis confirmed the high‐purity formation of Rh‐Pd composites, with no detectable impurity peaks (Figure 1A). Transmission electron microscopy (TEM) observations revealed that RhPd‐H exhibited irregular polyhedra with an average particle size of 16 ± 2 nm (Figure 1B). Elemental mapping performed via high‐angle annular dark‐field scanning transmission electron microscopy (HAADF‐STEM) showed highly uniform distribution and significant overlap of Rh and Pd signals within individual nanoparticles (Figure 1C), demonstrating clear co‐localization. This confirms the successful formation of an Rh‐Pd alloy structure rather than a core‐shell or physical mixture. Zeta potential measurements indicated that both rhodium‐palladium alloy (RhPd) and RhPd‐H carried negative charges in deionized (DI) water, with values of approximately −5.35 and −15.36 mV, respectively (Figure S1). The significantly higher electronegativity of RhPd‐H is likely attributed to the modulation of the surface electronic structure through hydrogenation, facilitating the adsorption of anions or hydroxyl groups. This feature contributes to enhanced colloidal stability in aqueous environments. We further assessed the colloidal behavior of RhPd‐H. As shown in Figure S2, the RhPd‐H nanoparticles maintained well stability in water, PBS and 1640 medium containing 10% FBS without obvious aggregation, supporting the reliability of subsequent biological evaluations. The biocompatibility of RhPd‐H was evaluated through a hemolysis assay. Fresh red blood cells were incubated with various concentrations of RhPd‐H nanoparticles, and the absorbance of the supernatant was measured at 540 nm. Results showed that the hemolysis ratio remained below 3% even at 60 µg/mL (Figure S3), providing a critical safety basis for the intravenous administration and in vivo application of the material.

### 2.2 Evaluation of Photothermal Triggered Hydrogen Release

The photothermal heating performance of RhPd‐H NPs demonstrated significant concentration‐ and power‐dependent behavior (Figure 1D,E). Under irradiation with a NIR laser (808 nm, 1 W/cm^2^), the equilibrium temperature increase of the solution rose from a 24.9 to 51.2°C as the concentration increased from 20 to 100 µg/mL. Similarly, at a fixed concentration (60 µg/mL), the temperature increase showed a linear growth with laser power (0.8‐1.2 W/cm^2^), reaching a maximum of ΔT ≈ 37.3 °C. In a supplementary experiment at a moderate concentration of 40 µg/mL under the same irradiation conditions (1 W/cm^2^, 5 min), RhPd exhibited a similar trend, confirming its effectiveness as a photothermal conversion core (Figure S4). Moreover, the material exhibited excellent photothermal stability. Over four consecutive cycles of “laser on for 10 min‐laser off for natural cooling for 10 min”, the maximum temperature difference for RhPd‐H in each cycle was less than 5.6 °C, and the heating/cooling curves were highly reproducible (Figure 1F). This indicates that the microstructure and optical properties of the materials remained largely unchanged after multiple photothermal cycles, demonstrating well photo‐thermal stability. Based on the heating and cooling curves over a 10‐min period, the photothermal conversion efficiency of RhPd‐H was calculated to be as high as η ≈ 65.18% (Figure S5), which surpasses that of most reported noble metal‐based photothermal materials. This high energy conversion capability is primarily attributed to the localized surface plasmon resonance (LSPR) effect of the RhPd alloy nanostructure and its broad absorption in the first NIR window, underscoring its significant potential for photothermal therapy applications.

Optical microscopy revealed the generation of numerous microbubbles in an aqueous solution of RhPd‐H nanoparticles under NIR irradiation, whereas no bubbles were observed under identical conditions in RhPd nanoparticles (Figure S6). Furthermore, MB reduction assays confirmed that only RhPd‐H nanoparticles produced reductive H_2_ gas upon laser exposure, leading to efficient bleaching of the dye. Quantitative results showed a 50.61% decrease in absorbance at 664 nm after 60 min of irradiation, corresponding to a cumulative hydrogen release of 32.9 µM. In contrast, RhPd nanoparticles induced almost no reduction of MB (Figure 1G). These control experiments effectively exclude photocatalytic water splitting as a major contributor, suggesting that the NIR‐triggered hydrogen release from RhPd‐H is driven primarily by photothermal decomposition of the hydride lattice. To further investigate the release mechanism, we examined the correlation between temperature rise and hydrogen evolution by varying laser power. A synchronous increase in both solution temperature and hydrogen generation with increasing laser power directly confirmed that the photothermal effect is the key driver for hydrogen release (Figure 1I). Thus, localized heating from photothermal conversion disrupts metal‐hydrogen bonds in RhPd‐H, promoting desorption and recombination of lattice hydrogen into H_2_ molecules [34]. Moreover, the NIR‐triggered hydrogen release from RhPd‐H exhibited high controllability under repeated laser on/off cycling. Using alternating 808 nm laser exposure (5 min ON/OFF), reproducible H_2_ generation occurred exclusively during irradiation phases and ceased promptly when the laser was turned off. This repeatable on/off behavior demonstrates the precise and reliable photo‐responsiveness of RhPd‐H for controlled H_2_ release (Figure 1H).

At the cellular level, when 4T1 cells pre‐stained with MB were treated with RhPd‐H and irradiated for 5 min, pronounced intracellular fading accompanied by a 90% reduction in fluorescence within 60 min was observed, indicating effective photothermal‐hydrogen release and hydrogen‐mediated reduction within cells (Figure 1J,K). By contrast, no significant fluorescence reduction was observed in any of the control groups. These results collectively establish that RhPd‐H enables controllable and bio‐reductive hydrogen release under NIR light through a mechanism reliant on the photothermal conversion of the RhPd core and thermal decomposition of palladium hydride, offering a robust tool for hydrogen‐based biological regulation.

### 2.3 Mechanism of Hydrogen Enhanced Intrinsic POD‐Mimetic Catalysis

**FIGURE 2:**
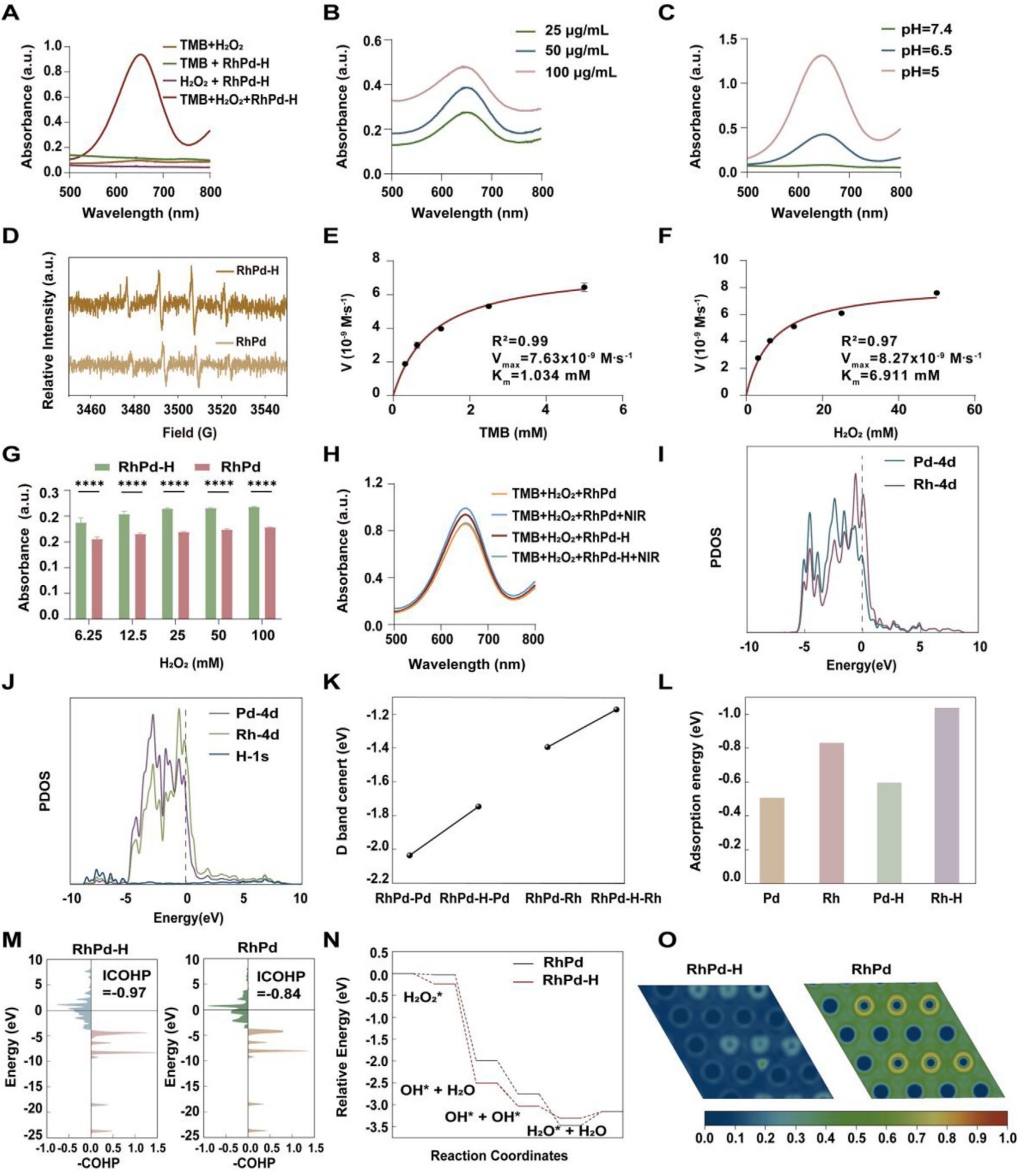
Hydrogen enhanced POD‐mimicking activity and DFT simulation. (A‐C) POD‐like activity of RhPd‐H in a concentration and pH‐dependent manner. (D) ESR spectra of RhPd‐H and RhPd NPs after adding H_2_O_2_ in pH 6.5 buffer. RhPd‐H nanozyme Michaelis‐Menten kinetics using (E) TMB and (F) H_2_O_2_ as the substrates. (G) Comparison of POD‐like activity of RhPd‐H and RhPd with different concentration of H_2_O_2_ (*n* = 3). (H) Comparison of POD‐like activity of RhPd‐H and RhPd ±NIR. The PDOS of RhPd (I) and RhPd‐H NPs (J). (K) The d‐band centers of the Pd and Rh sites. (L) The adsorption energy of H_2_O_2_ in RhPd and RhPd‐H NPs. (M) COHP for RhPd‐H and RhPd NPs. (N) Corresponding free energy diagrams of RhPd‐H and RhPd NPs for POD‐like reaction. (O) The electron localization functions on the surfaces of RhPd‐H and RhPd NPs. Statistical significance was determined by a two‐tailed Student's t‐test: ^*^ *p* < 0.05, ^**^ *p* < 0.01, ^***^ *p* < 0.001, ^****^ *p* < 0.0001.

Under acidic conditions at pH 6.5, RhPd‐H nanoparticles exhibited pronounced POD‐mimicking activity (Figure 2A). They catalyzed the decomposition of H_2_O_2_ to generate·OH which oxidized the substrate TMB to form a blue oxidized product (oxTMB) with a characteristic absorbance peak at 625 nm in a concentration‐dependent manner (Figure 2B). The catalytic activity was strongly pH‐dependent, with the highest efficiency observed at pH 5 and a progressive decrease at pH 6.5 and 7.4 (Figure 2C). This pH‐responsive behavior aligns with the acidic microenvironment preference of natural peroxidases, indicating the potential of RhPd‐H for targeted catalytic applications in acidic tumor regions. The kinetic analysis of the RhPd‐H nanozyme was conducted by varying the concentrations of H_2_O_2_ and TMB at pH 6.5. The Michaelis‐Menten model and double‐reciprocal plot were applied to determine the corresponding kinetic parameters. The Michaelis‐Menten constant (K_m_) and maximum reaction velocity (V_max_) for TMB were 1.034 mM and 7.63×10^−9^ M·s^−^^1^, respectively (Figure 2E). For H_2_O_2_, the K_m_ and V_max_ values were 6.911 mM and 8.27×10^−9^ M·s^−^^1^ (Figure 2F). Electron paramagnetic resonance (EPR) spectroscopy further confirmed ·OH generation, as evidenced by characteristic DMPO‐·OH signals in both RhPd‐H and RhPd systems (Figure 2D). Based on the results from UV–vis absorption spectroscopy and EPR characterization, RhPd‐H exhibited superior POD‐mimicking activity compared to RhPd at an equivalent mass concentration. Furthermore, evaluation across a range of H_2_O_2_ concentrations consistently demonstrated that the POD‐like activity of RhPd‐H surpassed that of RhPd under all tested conditions (Figure 2G). These findings reveals that the hydrogen atoms residing within the metal lattice effectively enhance the POD‐mimicking catalytic activity.

Upon NIR irradiation, the behaviors of the two materials diverged. The POD‐like activity of RhPd increased due to photothermally accelerated reaction kinetics, leading to a rise in oxTMB absorbance. For RhPd‐H, the released hydrogen consumed a portion of the in situ‐generated ·OH radicals, thereby partially offsetting the acceleration from photothermal heating and resulting in a slight decrease in oxTMB signal (Figure 2H).

To further investigate the mechanism underlying the hydrogen‐enhanced intrinsic POD‐mimetic catalysis, density functional theory (DFT) calculations were performed. The electronic structures of RhPd and RhPd‐H catalysts were elucidated by comparing their projected density of states (PDOS). As shown in Figure 2I, for RhPd, the d‐bands of Pd and Rh exhibit broad and continuous peak distributions. Orbital hybridization between H‐1s and Pd/Rh 4d orbitals confirms the formation of stable chemical bonds in RhPd‐H (Figure 2J). Concurrently, hydrogen incorporation sharpens the metal d‐bands and raises the Fermi level (Ef), enhancing electrochemical activity. As a result, these electronic modulations significantly improve the peroxidase‐like performance of RhPd metalenes. Furthermore, hydrogen incorporation upshifts the d‐band centers of both Pd and Rh: from −2.04 to −1.75 eV for Pd, and from −1.407 to −1.1 eV for Rh (Figure 2K). This enhancement boosts electroactivity and strengthens H_2_O_2_ adsorption. Adsorption energy calculations (Figure 2L) confirm this trend. RhPd‐H binds H_2_O_2_ more strongly than RhPd, as evidenced by its more negative adsorption energy. Between the two metal sites, Rh exhibits superior H_2_O_2_ affinity, positioning it as the primary active center for H_2_O_2_ capture during catalysis.

For verifying the binding strength between Rh sites and H_2_O_2_, the crystal orbital Hamiltonian population (COHP) was calculated (Figure 2M). RhPd‐H displays a markedly stronger interaction with H_2_O_2_ than RhPd, as evidenced by its superior bonding states. This is also supported by the more negative integrated COHP (ICOHP) value (−0.97 eV) for RhPd‐H. Based on the POD‐like reaction mechanism, the free energy changes of key elementary steps were computed (Figure 2N). The results showed that RhPd‐H is energetically more favorable than RhPd for the reduction of H_2_O_2_. The H_2_O desorption step in the POD‐like reaction favors RhPd‐H over RhPd, requiring only 0.28 V compared to 0.46 V. Moreover, the electron localization function (ELF) analysis on the surfaces of RhPd‐H and RhPd NPs revealed that hydrogen incorporation leads to a significant reduction in the charge on surface metal atoms (Figure 2O). This results in fewer electrons transferred from RhPd‐H to H_2_O, weakening H_2_O adsorption and thereby facilitating the subsequent catalytic cycle. In summary, the introduction of hydrogen atoms into RhPd metalenes can modulate their electronic properties, promote H_2_O_2_ adsorption, optimize the binding of reaction intermediates, and lower the energy barrier for water desorption, collectively enhancing the POD‐mimetic catalytic activity.

Given that Pd‐H materials typically exhibit both catalase (CAT)‐ and superoxide dismutase (SOD)‐like activities, yet our experiments show that RhPd‐H possesses neither, we performed theoretical calculations to analyze the free energy profiles of these two reaction pathways on Pd‐H and RhPd‐H surfaces. The superoxide anion (·O_2_^−^) acts as a Brönsted base and readily abstracts a proton from water to form hydroperoxyl (OOH), which was therefore selected as the starting reactant for evaluating SOD‐like activity. As shown in Figure S7A, Pd‐H binds OOH more weakly than RhPd‐H does. In the SOD‐like catalytic cycle, OOH must undergo further protonation or disproportionation. However, the overly strong binding of OOH on RhPd‐H stabilizes the intermediate and impedes subsequent conversion, leading to the loss of SOD‐like activity. For CAT‐like activity, the desorption of H_2_O_2_ and O_2_ from Pd‐H is more favorable than from RhPd‐H (Figure S7B), indicating that product desorption is hindered on RhPd‐H, which prevents active‐site regeneration and accounts for its lack of CAT‐like activity. These results reveal that Rh incorporation selectively modulates nanozyme activity. Although Pd‐H inherently exhibits POD‐, CAT‐, and SOD‐like activities, the introduction of Rh enhances only POD‐like activity while suppressing the other two. This exclusive retention of POD‐like activity enables RhPd‐H to achieve higher catalytic efficiency by avoiding the multifunctional competition that often compromises the performance of nanozymes with multiple activities.

### 2.4 Hydrogen‐Programmed Self‐Sustaining Intracellular Oxidative Storm

**FIGURE 3:**
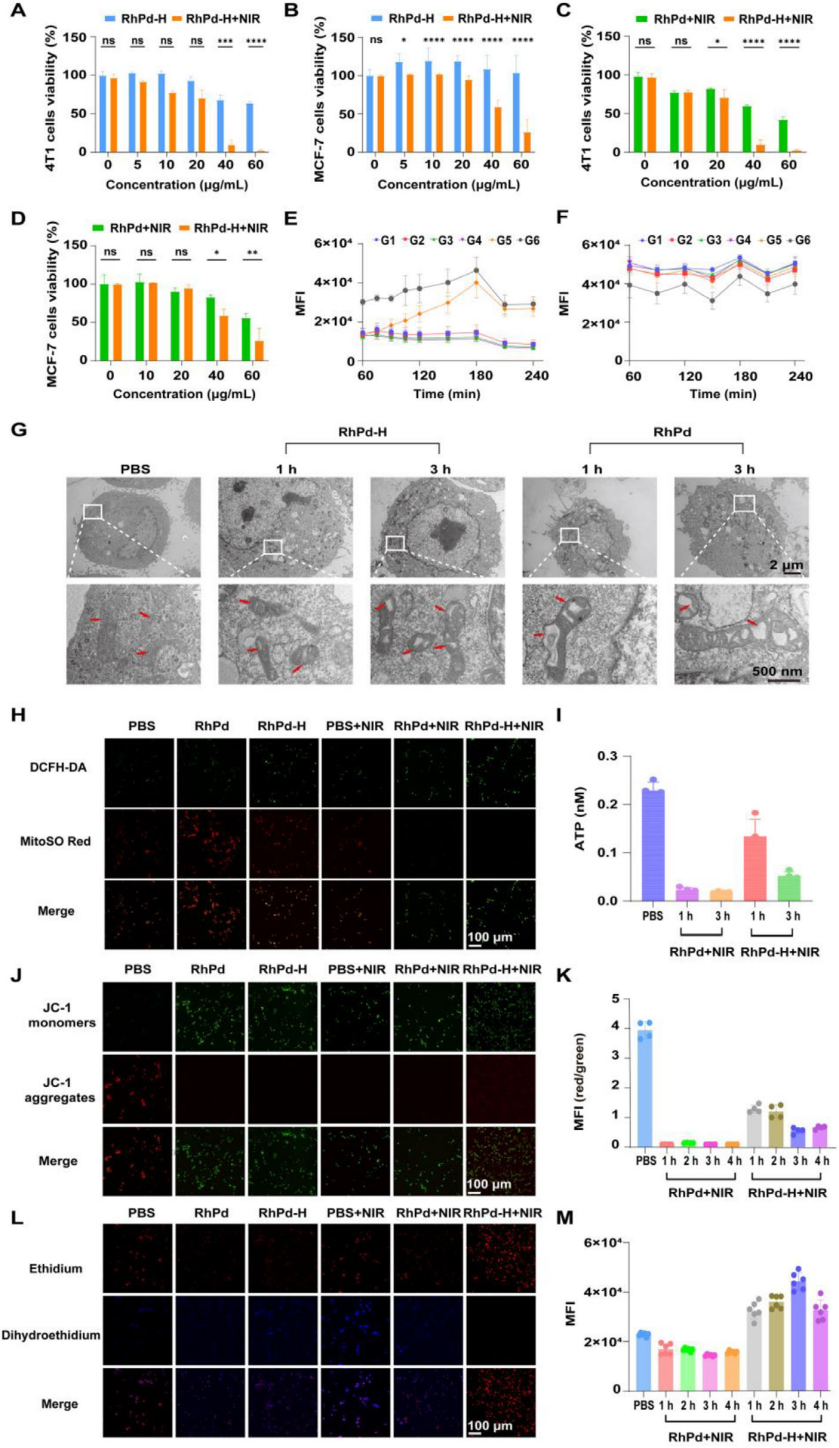
Mechanism of the sustained oxidative storm induced by RhPd‐H nanoenzyme. (A‐D) Viability of (A, C) 4T1 and (B, D) MCF‐7 cells treated with RhPd or RhPd‐H at varying concentrations (A, B) without and (C, D) with NIR irradiation (*n* = 3). Time‐dependent changes in the average fluorescence intensity of (E) DCFH‐DA and (F) MitoSOX Red under different treatments (*n* = 5). Treatment groups: G1, PBS; G2, RhPd; G3, RhPd‐H; G4, PBS+NIR; G5, RhPd+NIR; G6, RhPd‐H+NIR. (G)TEM images of mitochondria structures in 4T1 cells treated with RhPd+NIR and RhPd‐H+NIR at specific intervals (1 and 3 h). (H) DCFH‐DA/ MitoSOX Red double staining of 4T1 cells under different treatments (G1‐G6) at time of 2 h. (I) Detection of ATP levels in 4T1 cells under RhPd+NIR and RhPd‐H+NIR treatments at different time intervals (1 and 3 h) (*n* = 3). (J, L) CLSM images of 4T1 cells stained with (J) JC‐1 and (L) DHE under various treatments at time of 2 h. (K, M) Time‐dependent changes in the average fluorescence intensity of (K) JC‐1 red/green ratio (*n* = 4) and (M) DHE (*n* = 6) under RhPd+NIR and RhPd‐H+NIR treatments at different time intervals (1, 2, 3, and 4 h). Statistical significance was determined by a two‐tailed Student's t‐test: ^*^ *p* < 0.05, ^**^ *p* < 0.01, ^***^ *p* < 0.001, ^****^p < 0.0001.

The cytotoxicity of RhPd‐H against 4T1 and MCF‐7 cells was systematically evaluated using the CCK‐8 assay under varying concentrations and incubation durations. As shown in Figure 3A, RhPd‐H exhibited a time‐ and concentration‐dependent increase in toxicity toward 4T1 cells. After 24 h of co‐incubation at a concentration of 40 µg/mL, the viability of 4T1 cells decreased to 68%, suggesting that RhPd‐H‐mediated nano‐catalysis therapy induces cytotoxicity in this cell line. In contrast, under the same conditions, RhPd‐H showed no significant toxicity toward MCF‐7 cells. We further assessed the phototoxicity of RhPd‐H in vitro under 808 nm laser irradiation. Upon laser exposure, RhPd‐H demonstrated marked concentration‐dependent cytotoxicity in both 4T1 and MCF‐7 cells. When the concentration of RhPd‐H was increased to 40 µg/mL, the cell viability of 4T1 and MCF‐7 cells decreased to 9.72% and 58.99% (Figure 3A,B), respectively. To further investigate the role of hydrogen in enhancing the anticancer efficacy of the material, we compared the effects of RhPd and RhPd‐H on 4T1 and MCF‐7 cells under 808 nm laser irradiation across a range of concentrations. As depicted in Figure 3C,D, both materials induced concentration‐dependent cytotoxicity in the two cancer cell lines. Notably, RhPd‐H demonstrated superior anti‐tumor efficacy to RhPd within the cytotoxic concentration range in a dose‐dependent manner, with the advantage becoming increasingly pronounced at higher concentrations. For instance, at 40 µg/mL, the viability of 4T1 cells treated with RhPd‐H was only 16.31% of that in the RhPd‐treated group. These results indicate that hydrogen incorporation significantly enhances the combined therapeutic outcome of photothermal and nanocatalytic therapy, and this enhancement becomes particularly evident above a certain concentration threshold.

For investigating the synergistic antitumor mechanism of RhPd‐H NPs under NIR irradiation, we systematically evaluated their effects on intracellular ROS levels and mitochondrial function in 4T1 tumor cells. Detection of total intracellular ROS using the 2,7‐dichlorofluorescein diacetate (DCFH‐DA) probe revealed that compared to the PBS control group, after 3 h, RhPd + NIR treatment increased fluorescence intensity by approximately 2.77‐fold, while the RhPd‐H + NIR group exhibited a further increase to about 3.20‐fold (Figure 3E). This indicates that RhPd‐H + NIR induces more substantial cytosolic ROS accumulation. Using the MitoSOX Red mitochondrial superoxide indicator (MitoSOX Red), we next quantified mitochondrial ·O_2_^−^, a predominant ROS originating from the mitochondrial electron transport chain. The RhPd‐H + NIR group exhibited an approximately 14.16% reduction in fluorescence signal compared to the RhPd + NIR group (Figure 3F), which implies an obvious decrease in mitochondrial oxidative stress. DCFH‐DA/ MitoSOX Red double staining of 4T1 cells experiment further confirmed that RhPd‐H + NIR simultaneously induces cytosolic ROS elevation (DCFH‐DA fluorescence increased ∼8.49‐fold) and mitochondrial ROS suppression (MitoSO Red signal decreased ∼89%), demonstrating its capacity for spatially differential ROS modulation (Figure 3H). To delve deeper into the mitochondrial functional status underpinning the differential ROS modulation, we first examined the ultrastructural integrity of mitochondria via TEM (Figure 3G). Compared to the PBS control group with well‐defined and intact cristae, mitochondria in both the RhPd + NIR and RhPd‐H + NIR groups exhibited varying degrees of vacuolation, indicative of swelling and structural damage. This morphological impairment was both time‐dependent and hydrogen‐modulated. At both 1 and 3 h time points post‐treatment, the extent of vacuolation was consistently and markedly less severe in the RhPd‐H + NIR group than in the hydrogen‐free RhPd + NIR group. Furthermore, the damage progressed over time, with 3 h samples showing more pronounced vacuolation than their 1 h counterparts for both materials. This visual evidence directly demonstrates that hydrogen confers a incomplete preservation of mitochondrial architecture under stress, providing a morphological basis for the subsequent functional observations.

We then assessed the functional consequences of this compromised structure. The assessment of mitochondrial membrane potential (ΔΨm) using the JC‐1 probe revealed distinct kinetic profiles. Compared to the PBS group, the RhPd + NIR treatment induced an abrupt ΔΨm collapse, marked by an approximately 39‐fold decrease in the red/green fluorescence ratio within 1 h. In contrast, the RhPd‐H + NIR group exhibited a more gradual decline. While a significant decrease was observed as early as 1 h, the ΔΨm in this group continued to diminish progressively at the 3‐ and 4‐h time points. At the 3 h mark, the fluorescence ratio in the RhPd‐H + NIR group was 7.1‐fold higher than that in the RhPd + NIR group (Figure 3K). Confocal laser scanning microscope (CLSM) imaging at 2 h post‐treatment provided direct visual evidence for this delayed collapse: the RhPd‐H + NIR group exhibited distinct red fluorescence, whereas only green fluorescence was observed in the RhPd + NIR group. These collective findings unequivocally underscore the role of hydrogen in decelerating the loss of ΔΨm (Figure 3J). Furthermore, intracellular ATP level measurements corroborated this state of dysfunctional yet sustained activity of mitochondria. At the 1 h time point, the ATP level in the RhPd‐H + NIR group was significantly lower than that of the PBS control (approximately 58% of the control level) but substantially higher than that in the ATP‐depleted RhPd + NIR group (approximately 5.8‐fold of the RhPd + NIR group). This indicates that despite failing to produce energy efficiently, the mitochondria in the RhPd‐H + NIR group remained metabolically active and were struggling to function. By 3 h, the ATP level in the RhPd‐H + NIR group had declined markedly to about 22.7% of the control level, yet remained 2.7‐fold higher than that in the RhPd + NIR group, illustrating the eventual failure of this compromised state but a clear delay in energetic collapse compared to the hydrogen‐free treatment (Figure 3I). Despite its partial protective effects on mitochondria, hydrogen is unable to fully counteract the underlying dysfunction of the electron transport chain. Consequently, the cells exhibit significant production of ·O_2_^−^ stemming from this impairment. The RhPd‐H + NIR treatment triggered a substantial increase in endogenous superoxide, as evidenced by confocal imaging with Dihydroethidium (DHE), which showed an approximately 6.8‐fold higher ·O_2_^−^ fluorescence intensity compared to the RhPd + NIR group (Figure 3L,M). This observation confirms the sustained generation of ·O_2_^−^, with mitochondria as a primary source.

Based on these experimental findings, we propose that RhPd‐H NPs exert potent tumor cell killing via a hydrogen‐mediated mitochondrial function reprogramming mechanism. Under NIR irradiation, RhPd‐H concurrently delivers PTT, nano‐catalysis and hydrogen release. PTT and nano‐catalysis act synergistically in the cytosol to generate a burst of ·OH. Simultaneously, the released hydrogen diffuses into mitochondria and scavenges ROS, thereby mitigating mitochondrial oxidative damage and maintaining membrane potential stability. Unlike conventional photothermal and catalytic strategies that rapidly induce mitochondrial destruction, the protective action of hydrogen allows mitochondria to enter a distinct state of preserved structural integrity but compromised function, as evidenced by intermediate ATP production. The decrease in electron transport chain efficiency increases electron leakage, leading to continuous superoxide generation and effectively transforming mitochondria into persistent endogenous ROS generators. Notably, this hydrogen‐mediated mitochondrial modulation operates in concert with an additional pro‐oxidant pathway. It has been established that H_2_ can be catalytically converted to carbon monoxide (CO) in the TME, initiating rapid pro‐oxidant signaling [35]. Rather than conflicting with our observations, this CO‐mediated mechanism complements the mitochondrial reprogramming we describe. We propose a synergistic framework: the rapid generation of CO provides an initial acute oxidative trigger, while the hydrogen‐induced alteration of mitochondrial redox kinetics establishes a prolonged state of dysfunctional activity. Instead of undergoing immediate collapse, the compromised mitochondria sustain heightened metabolic and oxidative stress over time. Thus, these two pathways of rapid CO signaling and sustained mitochondrial dysfunction work in concert to create a more devastating and persistent oxidative storm. Consequently, their synergistic action sustains elevated total intracellular ROS levels and significantly prolongs oxidative stress, ultimately overwhelming cellular antioxidant repair capacity and inducing irreversible lipid peroxidation and cell death. This shift in strategy from acute oxidative shock to a sustained oxidative storm significantly enhances therapeutic thoroughness and efficacy, providing a new perspective for developing next generation synergistic tumor treatment platforms.

### 2.5 Bio‐Interface Engineering of PVP‐Modified RhPd‐H Nanoparticles: Antifouling, Long‐Circulating and Active‐Targeting Behaviors

**FIGURE 4:**
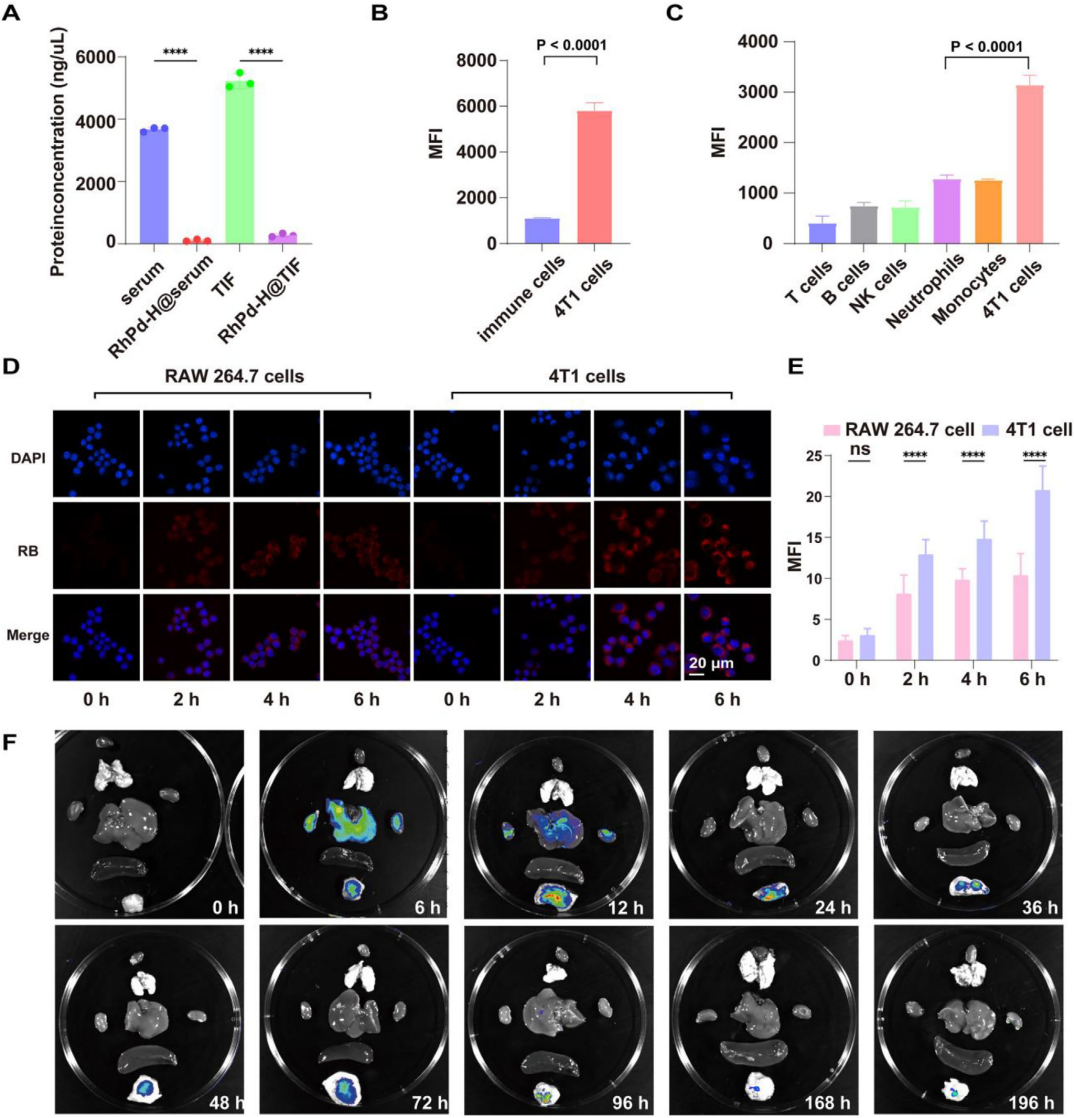
Antifouling properties, immune evasion, and tumor targeting of RhPd‐H. (A) Quantification of protein adsorption on RhPd‐H in serum and tumor interstitial fluid (*n* = 3). (B) Comparison of ability of RhPd‐H uptake between total immune cells in peripheral blood and 4T1 cells (*n* = 4). (C) Comparison of ability of RhPd‐H uptake between various types of immune cells and 4T1 cells (*n* = 4). (D, E) CLSM fluorescence images (D) and quantification results (E) of RAW 264.7 and 4T1 cells incubated with RB‐labeled RhPd‐H at different time intervals (0, 2, 4, and 6 h) (*n* = 25). (F) Ex vivo fluorescence imaging showing the distribution and retention of RB‐labeled RhPd‐H in mice. Statistical significance was determined by two‐tailed Student's t‐test: ^*^ *p* < 0.05, ^**^ *p* < 0.01, ^***^ *p* < 0.001, ^****^ *p* < 0.0001.

The exceptional distribution behavior of the RhPd‐H NPs in vivo is closely linked to the cascade of biological effects mediated by its surface properties. BCA protein adsorption assays confirmed that polyvinyl pyrrolidone (PVP) modification confers significant anti‐fouling capability to the material in both serum and tumor interstitial fluid, effectively suppressing the formation of a protein corona (Figure 4A). This property originates from a dense hydration layer constructed by PVP chains via steric hindrance and hydrogen bonding, which specifically blocks the adsorption of key opsonins and other plasma proteins that mediate immune recognition [36, 37]. This molecular‐level stealth characteristic directly dictates subsequent cellular interactions. To dynamically evaluate cellular uptake, we labeled the nanoparticles with Rhodamine B (RB) and quantified their internalization using confocal microscopy over a period of 0, 2, 4, and 6 h (Figure S8; Figure 4D). The results exhibited a profoundly divergent uptake kinetics between macrophages and 4T1 tumor cells. Macrophages exhibited minimal phagocytosis across all time points with fluorescence intensity remaining at consistently low levels, revealing successful evasion of immune surveillance. In contrast, 4T1 tumor cells demonstrated a rapid, time‐dependent accumulation of nanoparticles. This trend was further quantified through confocal image analysis, which showed the disparity becoming progressively more evident over time. Ultimately, at the 6 h time point, the fluorescence intensity was 2‐fold higher than in macrophages (Figure 4E), serving as dynamic visual evidence for the cellular preference. For directly comparing the cellular uptake preferences between tumor cells and various immune cells under physiologically competitive conditions, we established two precision co‐culture models for parallel analysis. In the “total competition” model, 4T1 tumor cells were paired with an equal number of peripheral blood leukocytes. Flow cytometry analysis revealed that after incubation with RB‐labeled RhPd‐H NPs, the mean fluorescence intensity (MFI) of 4T1 cells was approximately 6‐fold higher than that of the total immune cell population (Figure 4B). To more precisely investigate the primary phagocytic forces in circulation, we established a targeted competition model by equalizing the number of 4T1 cells and neutrophils. Strikingly, although neutrophils and eosinophils were confirmed as the most active phagocytic subsets among immune cells, their nanoparticle uptake efficiency remained significantly lower than that of 4T1 tumor cells. These findings demonstrate that the nanoparticles not only evade clearance by key phagocytic systems including neutrophils and monocytes/macrophages but are also actively and efficiently internalized by tumor cells (Figure 4C). This distinct cellular behavior characterized by simultaneous immune evasion and preferential tumor targeting constitutes a fundamental mechanism underlying efficient tumor‐directed delivery.

The significantly enhanced uptake efficiency observed in 4T1 cells can be attributed to the synergistic effect between their intrinsically high endocytic activity and the surface modification with PVP. Since macrophages and neutrophils primarily rely on opsonin‐dependent phagocytosis, PVP coating effectively evades their recognition and clearance. Meanwhile, tumor cells often overexpress endocytic receptors such as scavenger receptor class B (SR‐B) which are capable of directly recognizing nanoparticles under a low‐protein‐corona state. The high‐concentration PVP surface modification effectively prevents nonspecific protein adsorption. Furthermore, the formed hydrophilic polymer layer may actively promote nanoparticle‐receptor binding by optimizing spatial conformation and interfacial interactions [38, 39]. The co‐culture experiment definitively demonstrates that this cellular preference is not merely a result of disparate culture conditions, but a robust competitive advantage for tumor cells in a more physiologically relevant setting where both cell types are present. This favorable cellular preference is critical for enhancing the bioavailability of the nanoparticles for tumor targeting and forms the cellular basis for the well in vivo distribution pattern.

The highly efficient cellular uptake by tumor cells, as conclusively demonstrated above, provides a crucial mechanistic explanation for the outstanding in vivo tumor accumulation and prolonged retention (Figure 4F). The RhPd‐H NPs exhibited a remarkably sustained presence within the tumor, with a detectable signal persisting for up to 196 h post‐injection. This extraordinarily long tumor retention can be attributed to a powerful synergistic mechanism: the long‐lasting systemic circulation caused by immune evasion, followed by active and efficient cellular internalization into tumor cells. This two‐step process including extravasation into the tumor interstitium and actively “trapped” inside tumor cells, creates a formidable barrier against nanoparticle washout, effectively sequestering them within the tumor mass for an extended duration. Conversely, the systemic safety and efficient clearance profile are equally noteworthy. The fluorescence signals in all major normal organs including the heart, liver, spleen, lungs, and kidneys diminished to negligible levels by 24 h, revealing a rapid and comprehensive clearance from the body. This distinct biodistribution profile characterized by prolonged tumor retention coupled with rapid systemic clearance holds profound implications for both therapeutic efficacy and biosafety. From a safety perspective, the minimal long‐term accumulation in vital organs significantly reduces the risk of off‐target toxicity and potential chronic damage, a common concern with many nanomedicines. This combination of a durable tumor‐resident effect and a clean body clearance mechanism underscores the superior in vivo performance and clinical translation potential of our PVP‐coated RhPd‐H NPs.

### 2.6 In Vivo Therapeutic Evaluation

**FIGURE 5:**
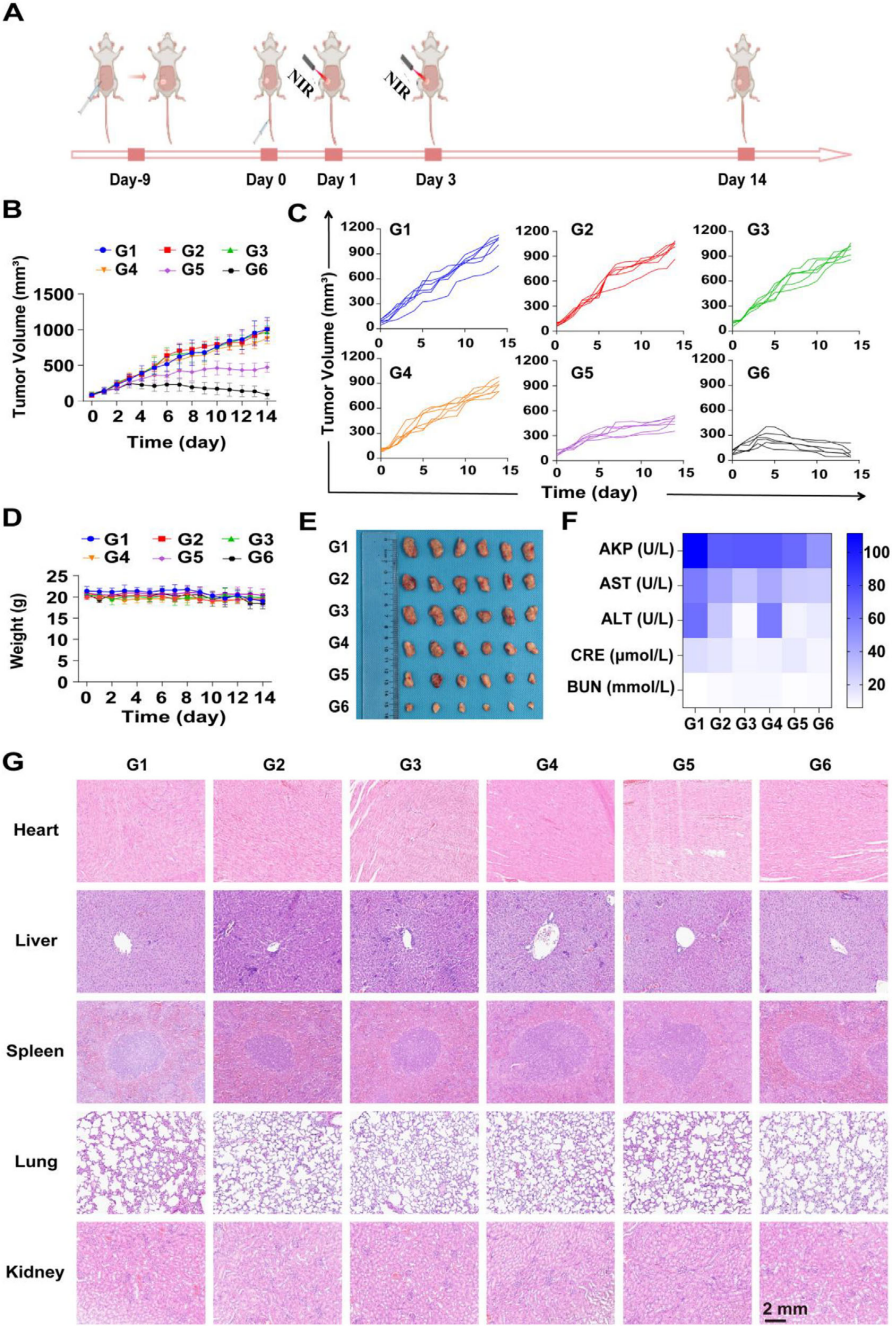
In vivo therapeutic efficacy and biosafety evaluation of RhPd‐H. (A) Schematic illustration of the experimental timeline and treatment protocol. (B) Tumor growth curves monitored over the treatment period (*n* = 6). (C) Individual growth curves of 4T1 tumors after various treatments. (D) Body weight changes in mice during the treatment course (*n* = 6). (E) Representative photographs of excised tumors from each treatment group (*n* = 6). (F) Heat map of hematological and biochemical parameters of blood samples from differently treated mice. (G) H&E‐stained sections of major organs. Treatment groups: G1, PBS; G2, RhPd; G3, RhPd‐H; G4, PBS+NIR; G5, RhPd+NIR; G6, RhPd‐H+NIR. Statistical significance was determined by two‐tailed Student's t‐test: ^*^ *p* < 0.05, ^**^ *p* < 0.01, ^***^ *p* < 0.001, ^****^ *p* < 0.0001.

In the in vivo therapeutic assessment (Figure 5A), mice bearing 4T1 tumors were randomly allocated into six groups: PBS, RhPd, RhPd‐H, PBS + NIR, RhPd + NIR and RhPd‐H + NIR. When tumor volumes reached approximately 100 mm^3^, the respective formulations were administered via tail vein injection at a uniform dose of 5 mg/kg. At 24 h post‐injection, tumors in the NIR groups were irradiated with an 808 nm laser (1 W/cm^2^, 5 min), and the irradiation was repeated after 48 h to reinforce the therapeutic effect. Significantly, since laser irradiation triggers partial hydrogen release from RhPd‐H, we assessed whether this affects its POD‐like activity under our treatment conditions. After two cycles of laser irradiation, RhPd‐H retained robust POD‐like activity with only a slight decrease, remaining significantly higher than that of RhPd (Figure S9). This sustained POD‐like activity ensured the therapeutic efficacy of RhPd‐H under our treatment regimen. Tumor volumes and body weights were monitored every two days over a 14‐day period to evaluate treatment efficacy and systemic toxicity. The therapeutic outcomes revealed a clear and stepwise enhancement in antitumor efficacy across the combination therapy groups (Figure 5B,C,E). As anticipated, the groups receiving RhPd or RhPd‐H alone without laser irradiation showed only marginal tumor growth inhibition compared to the PBS control, suggesting that the catalytic activity of the nanoparticles in the absence of external activation is insufficient for effective tumor suppression. To further investigate the photothermal effect, we monitored the tumor temperature during NIR irradiation using an infrared thermal camera, with the RhPd + NIR and RhPd‐H + NIR groups reaching similar local temperatures of 52.9°C and 53.6°C, respectively (Figure S10). In the RhPd + NIR group, which combines the photothermal effect of RhPd with its catalytic function, a moderate but significant tumor suppression rate of approximately 53% was achieved. This result underscores the critical role of localized hyperthermia, which not only directly damages tumor cells but also enhances the catalytic reaction kinetics, leading to improved therapeutic outcomes. The RhPd‐H + NIR group representing the full triple‐combination therapy exhibited the most potent antitumor effect, achieving a remarkable tumor suppression rate of 91%. Of note, although the prolonged tumor retention of RhPd‐H could theoretically support additional treatments, we intentionally limited the regimen to two irradiations. This minimal yet sufficient schedule achieved optimal efficacy while minimizing potential safety risks to peritumoral normal tissues. This profound efficacy can be mechanistically attributed to a fundamental strategic shift enabled by hydrogen. Unlike the direct but transient oxidative shock from photothermal‐catalytic therapy alone, the introduced hydrogen actively reprograms mitochondrial function, transforming the organelles from ATP producers into persistent generators of endogenous superoxide. This action establishes a sustained two‐pronged ROS attack: a direct burst of exogenous ·OH from the cytosol combined with continuous endogenous ·O_2_^−^ from the mitochondria. This prolonged oxidative storm overwhelmingly surpasses cellular repair capacities, leading to irreversible damage and the superior tumor eradication efficacy.

Crucially, this potent therapeutic effect was achieved without eliciting significant systemic toxicity. Body weights remained stable across all groups throughout the treatment period, showing no notable fluctuations (Figure 5D). This safety profile is further corroborated by histological analysis (H&E staining) of major organs (heart, liver, spleen, lungs, and kidneys), which revealed no apparent pathological lesions or signs of damage (Figure 5G). The high biosafety is consistent with our earlier pharmacokinetic findings. The rapid clearance of the nanoparticles from all major organs within 24 h effectively prevents long‐term organ retention and minimizes the risk of off‐target toxicity. This “hit‐and‐run” profile where the nanoparticles exert their therapeutic effect in the tumor and are then efficiently cleared is a highly desirable characteristic for nanomedicines. In addition, comprehensive blood biochemical analysis post‐treatment showed that key markers of liver/kidney function including alkaline phosphatase (AKP), alanine aminotransferase (ALT), aspartate aminotransferase (AST), blood urea nitrogen (BUN) and creatinine (CRE) all remained within normal physiological ranges (Figure 5F), ensuring the well biocompatibility of our treatment strategy.

### 2.7 RhPd‐H Nanoplatform Reverses Immunosuppression and Elicits Systemic Anti‐Tumor Immunity

**FIGURE 6:**
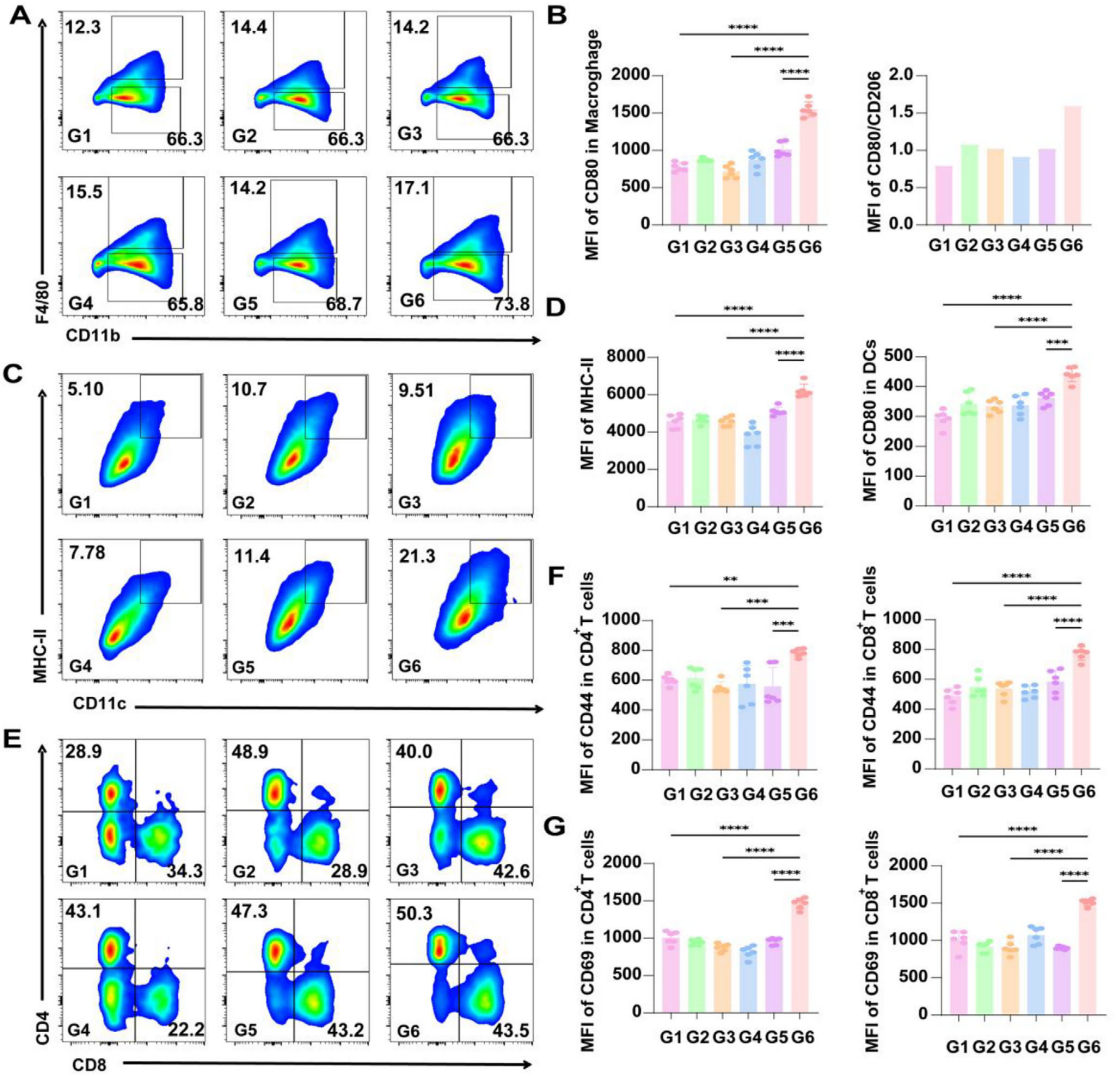
Activation of tumor‐specific immune responses. (A) Representative flow cytometry plots showing F4/80^+^/CD11b^+^ cells from different treatment groups. (B) Expression of macrophage‐associated markers: CD80 and CD80/CD206 (*n* = 6). (C) Representative flow cytometry plots showing MHC‐II^+^/CD11c^+^ cells from different treatment groups. (D) Expression of DCs‐associated markers: MHC‐II and CD80. (E) Representative flow cytometry plots showing CD4^+^/CD8^+^ cells from different treatment groups. (F) Quantitative analysis of T cell activation markers: CD44 and CD69 expression in CD4^+^ T cells; (G) CD44 and CD69 expression in CD8^+^ T cells (*n* = 6). Groups: G1, PBS; G2, RhPd; G3, RhPd‐H; G4, PBS + NIR; G5, RhPd + NIR; G6, RhPd‐H + NIR. Statistical significance was determined by two‐tailed Student's t‐test: ^*^ *p* < 0.05, ^**^ *p* < 0.01, ^***^ *p* < 0.001, ^****^ *p* < 0.0001.

To elucidate the immunological basis underlying potent tumor suppression by the combined photothermal, catalytic and hydrogen therapy, we systematically profiled the tumor immune microenvironment using flow cytometry. Compared with the PBS control and RhPd+NIR groups, RhPd‐H+NIR treatment induced a pronounced shift in macrophage polarization toward an anti‐tumor M1 phenotype (Figure 6A,B, gating methodology in Figure S11). The MFI of M1 type (CD80^+^) TAMs increased 1.99‐fold over the PBS group and 1.53‐fold over the RhPd+NIR group. Correspondingly, the M1/M2 ratio rose to 1.589 in the RhPd‐H+NIR group, significantly higher than that in the PBS (0.786) and RhPd+NIR (1.014) group, indicating a hydrogen‐enhanced anti‐tumor immune microenvironment. DCs maturation was also robustly potentiated by hydrogen release. The MFI of CD80 and MHC‐II in tumor‐draining lymph nodes was elevated by approximately 1.5 and 1.2‐fold, respectively, relative to the PBS group, and by 1.36 and 1.22‐fold over the RhPd+NIR group (Figure 6C,D, gating methodology in Figure S11), revealing the critical role of hydrogen in promoting antigen presentation and DC‐mediated T cell priming. This enhanced innate immune activation further translated to adaptive antitumor immunity. RhPd‐H+NIR treatment significantly increased tumor‐infiltrating T cell populations, with CD4^+^ T cells comprising 50.3% of total T cells, markedly higher than the 28.9% in the PBS group and 47.3% in RhPd+NIR group, while CD8^+^ T cells constituted 43.5%, compared to 34.3% and 43.2% in the respective control groups (Figure 6E, gating methodology in Figure S12). T cell activation was consistently enhanced in CD4^+^ T cells, CD44 and CD69 expression increased by approximately 1.3 and 1.45‐fold over PBS controls, and by 1.4 and 1.5‐fold over the RhPd+NIR group (Figure 6F). Similarly, CD8^+^ T cells exhibited 1.59 and 1.5‐fold upregulation in CD44 and CD69 relative to the PBS group, and 1.33 and 1.67‐fold increases over the RhPd+NIR group (Figure 6G). Collectively, these results highlight the indispensable contribution of hydrogen in amplifying the breadth and potency of antitumor immunity, bridging innate immune reprogramming to robust and durable T cell‐mediated responses.

Beyond localized immune activation within the TME, we observed a significant upregulation of the T cell activation marker CD44 in the spleen (Figure S13, gating methodology in Figure S14), a systemic immune organ. This finding suggests that the antitumor immune response elicited by RhPd‐H + NIR treatment is not confined to the tumor site but extends to the systemic level. It further implies that antitumor T cells may be differentiating toward effector phenotypes, indicating the initiation of a systemic immune response. In summary, the RhPd‐H nanoplatform reverses the immunosuppressive microenvironment locally by promoting DC maturation, enhancing T cell infiltration and activation, and reprogramming macrophage polarization. Furthermore, it induces preliminary systemic immune activation through pre‐activation of splenic T cells. The combination of these localized and systemic effects significantly enhances the potency and durability of the anti‐tumor immune response, providing a robust foundation for future applications in cancer immunotherapy.

## 3 Conclusion

In summary, this work successfully constructs a novel NIR‐responsive RhPd‐H nanoplatform that integrates nanocatalytic therapy, photothermal conversion and hydrogen release. The proposed nanoplatform demonstrates good tumor‐targeting capability and bioavailability, characterized by prolonged tumor retention and rapid clearance from normal organs, ensuring high therapeutic specificity and minimized systemic toxicity. More importantly, unlike traditional catalytic or photothermal therapies which rely on a short‐lived, and one‐time burst of reactive oxygen species (ROS), our platform triggers a sustained and self‐amplifying oxidative storm. This dual‐source storm is powered by both exogenous ·OH from photothermal‐enhanced catalysis and a continuous flow of endogenous ·O_2_^−^ from mitochondria reprogrammed by hydrogen. This shift from an acute oxidative shock to a prolonged oxidative assault effectively overwhelms the cellular antioxidant capacity, leading to irreversible tumor cell death. Furthermore, this potent therapeutic outcome triggers robust ICD, activates DCs and initiates a systemic antitumor immune response, thereby positioning RhPd‐H as a comprehensive platform for combinatory therapy. Consequently, it demonstrates markedly augmented catalytic therapeutic outcome through synergized metabolic intervention and immune activation, providing a new conceptual framework for designing advanced catalytic therapies centered on persistent oxidative disruption. While the RhPd‐H platform demonstrates promising therapeutic efficacy, this study has certain limitations. It is important to note that the current findings are primarily based on subcutaneous tumor models, which may not fully recapitulate the microenvironment of deeper or metastatic lesions. Furthermore, the long‐term biosafety and potential cumulative toxicity of the nanomaterials require more systematic investigation in future studies.

## 4 Experimental Method

### 4.1 Reagents

Palladium (II) acetylacetonate (98%) was purchased from Sigma‐Aldrich and Rh (III) acetylacetonate (97%) were obtained from Aladdin (China). Methylene blue (MB), RB were obtained from Aladdin (Shanghai, China). DCFH‐DA, DHE, MitoSOX Red, JC‐1 Kit, DiO, CCK‐8 were obtained from Beijing Solarbio Science & Technology (Beijing, China). Fetal bovine serum (FBS) was obtained from Gibco BRL (Carlsbad, CA, USA). The water used in all experiments was ultrapure. All reagents were used as received without further purification.

### 4.2 Evaluation of Photothermal Conversion Capability of RhPd‐H

The detailed synthesis of RhPd‐H and RhPd, including the hydrogen doping process and relevant characterizations, has been reported in our previous work [40]. The photothermal performance of RhPd‑H was assessed under three experimental conditions. First, solutions of RhPd‑H at varying concentrations (20, 40, 60, 80, and 100 µg/mL) were irradiated with an 808 nm laser at 1 W/cm^2^ for 5 min, and the temperature changes were recorded using an infrared thermal camera. Second, a 40 µg/mL RhPd‑H sample was exposed to 808 nm laser irradiation at different power densities (0.8, 1.0, and 1.2 W/cm^2^) for 5 min, while temperature variations were monitored in real time. Finally, the photothermal stability was evaluated by subjecting the 40 µg/mL sample to five repeated heating‑cooling cycles. Each cycle consisted of 5 min irradiation (808 nm, 1 W/cm^2^) followed by 20 min of natural cooling, with temperature continuously tracked by the thermal imager.

### 4.3 Peroxidase Reaction Assay

Under pH 6.0, a mixture containing TMB (0.4 mM), H_2_O_2_ (50 mM), and RhPd‐H nanozyme (50 µg/mL) were incubated. A control group consisting of TMB and H_2_O_2_ without the nanozyme was also prepared. The absorbance of each group was measured at a wavelength of 652 nm to evaluate the POD‐like activity of RhPd‐H. To investigate the effect of pH on catalytic activity, the reaction system with fixed reactant concentrations (TMB 0.4 mM, H_2_O_2_ 50 mM, and RhPd‐H 50 µg/mL) was adjusted to different pH values (5.0, 6.5, and 7.4). The absorbance changes at 652 nm were monitored to analyze the catalytic performance of the nanozyme under varying acidity conditions. Furthermore, to examine the influence of nanozyme concentration on its catalytic activity, different concentrations of RhPd‐H (25, 50, and 100 µg/mL) were incubated with TMB (0.4 mM) and H_2_O_2_ (50 mM) at pH 6.0. The absorbance at 652 nm was measured to systematically evaluate the trend of the catalytic reaction as a function of nanozyme concentration.

### 4.4 DFT Calculations

All DFT calculations were performed with the plane‐wave basis set as implemented in the Vienna Ab Initio Simulation Package (VASP) [41], and the electrons and ions interactions were described by the projector augmented wave (PAW) potential [42, 43]. The exchange‐correlation interactions were determined by the Perdew‐Burke‐Ernzerhof (PBE) functional within the generalized gradient approximation (GGA) [44]. The plane wave energy cutoff of 500 eV, and the convergence criterion for the residual force and energy was set to 0.05 eV Å^−1^ and 10−5 eV, respectively. The empirical correction in Grimme's method (DFT‐D3) was used to describe the van der Waals (vdW) interactions [45]. The Brillouin region was sampled by the Monkhorst‐Pack method with a 2 × 2 × 1 k‐point mesh. The change in the Gibbs free energy change (ΔG) for each possible step during the electrochemical synthesis of urea was obtained using the computational hydrogen electrode (CHE) model [46, 47]. The ∆G for all electrochemical steps was defined as: ΔG = ΔE + ΔEZPE − TΔS, where the reaction energy (ΔE) can be directly obtained by analyzing the DFT total energies. The zero‐point energy difference (ΔEZPE) between the products and the reactants can be computed from the vibrational frequencies. ΔS is the change in entropy between the products and the reactants at room temperature (T = 298.15 K).

### 4.5 Hydrogen Release Kinetics Assay

A standard curve was established using MB solutions at concentrations of 0, 3.125, 6.25, 12.5, 25, and 50 µg/mL by measuring the absorbance at 664 nm. To assess light‑triggered hydrogen release, 500 µL of RhPd‑H (1 mg/mL) was mixed with MB (final concentration: 50 µg/mL) in a 12‑well plate. The system was irradiated with an 808 nm laser (1 W/cm^2^), and the absorbance at 664 nm was monitored in real time using a UV‑Vis spectrophotometer. For release kinetics, the same system was subjected to three on/off cycles (5 min irradiation followed by 5 min rest) under 808 nm laser (1 W/cm^2^), with continuous absorbance recording. Hydrogen release was quantified based on the standard curve. Visual confirmation was obtained by irradiating 100 µL of RhPd‑H (1 mg/mL) in a 96‑well plate (808 nm, 1 W/cm^2^, 5 min), then transferring 10 µL to a glass slide for microscopic observation of bubble formation.

To obtain dual temperature‑increase and hydrogen‑release profiles under different power densities, 500 µL of RhPd‑H (1 mg/mL) containing MB (50 µg/mL) was irradiated with an 808 nm laser at 0, 0.3, 0.6, 0.9, 1.2, and 1.5 W/cm^2^ for 10 min. The solution temperature was recorded, and the absorbance at 664 nm was measured continuously until stabilization. The released hydrogen was calculated from the standard curve.

### 4.6 RhPd‐H Uptake of Co‐Cultured 4T1 and Immune Cells

4T1 cells were seeded in 12‐well plates at a density of 1×10^5^ cells/well. After 24 h of incubation, the serum was removed by washing three times with PBS. Carboxyfluorescein Succinimidyl Ester (CFSE) diluted at a ratio of 1:40000 was added, and the cells were stained at 37°C for 10 min. Following staining, the cells were washed three times with PBS, harvested by trypsinization, and collected as cell pellets by centrifugation. Peripheral blood was collected from Balb/c mice, anticoagulated with 50 µL of heparin sodium and used to resuspend the 4T1 cells to prepare a cell suspension containing both 4T1 cells and leukocytes. The cell suspension was incubated with RhPd‐H (RB‐labeled, 40 µg/mL) at a volume ratio of 1:1 (100 µL each) in a metal bath for 1 h (37°C, 500 rpm), with gentle flicking every 20 min. After incubation, all cells were collected by centrifugation at 450×g for 5 min to wash away unphagocytosed nanomaterials, and then resuspended in F‐PBS. Flow cytometry dyes were added for staining at room temperature for 15 min. The staining was terminated by adding 1 mL of 2% F‐PBS, and the cells were centrifuged at 450×g for 5 min. The resulting pellet was resuspended in 200 µL of PBS, filtered, and then subjected to flow cytometry analysis.

### 4.7 Cellular‐Level Assessment of Hydrogen‐Mediated Bio‐Reduction

4T1 tumor cells were cultured in RPMI‐1640 complete medium supplemented with 10% FBS and 1% penicillin–streptomycin at 37 °C under 5% CO_2_. Cells were seeded in a 96‐well plate at a density of 1×10^4^ cells per well and cultured for 24 h before treatment. Each well was incubated with 100 µL of complete medium containing 300 µg/mL MB for 1 h. After incubation, the medium was removed, and the cells were washed three times with PBS buffer. Then, 100 µL of complete medium containing 80 µg/mL RhPd or RhPd‐H was added to each well, while control groups received PBS alone. Following 5 min of 808 nm laser irradiation (1 W/cm^2^), the loss of blue color indicating MB reduction was observed under a microscope.

### 4.8 Preparation and Characterization of RhPd‐H@Serum Protein Corona

RhPd‐H solution (1 mg/mL) was mixed with healthy human serum at a volume ratio of 1:3 and incubated at 37°C for 1.5 h. After incubation, the mixture was centrifuged at 14 000 rpm for 10 min at 4°C to pellet the complexes. The supernatant was carefully discarded, and the precipitate was washed three times with pre‐chilled PBS buffer. The supernatant from the final wash was retained for further analysis. Finally, the precipitate was resuspended in pre‐chilled PBS to obtain the RhPd‐H@serum protein corona complexes. For protein quantification, 10 µL of RhPd‐H@serum protein corona complexes were mixed with 200 µL of BCA working solution (prepared at a ratio of reagent A to B = 1:50). The mixtures were incubated at 37°C for 30 min, and the absorbance at 562 nm (A_562_) was measured.

### 4.9 Preparation and Characterization of RhPd‐H@Tumor Antigen Corona Complexes

RhPd‐H was diluted to 50 µg/mL using the RPMI‐1640 complete medium. After co‐incubating 4T1 cells with RhPd‐H for 4 h, the treatment group was exposed to NIR laser irradiation (1 W/cm^2^, 10 min). The system was then returned to the cell culture incubator and incubated overnight. The culture medium was collected and centrifuged at 14 000 rpm for 10 min at 4°C to pellet any cells or debris. The supernatant was retained, and its protein content was quantified using the BCA assay. The pristine RhPd‐H nanoparticles were mixed with the obtained supernatant at a predetermined ratio and incubated at 37°C for 1.5 h. After incubation, the mixture was centrifuged at 14 000 rpm for 10 min at 4°C. The precipitate was washed three times with ice‐cold PBS buffer and finally resuspended in ice‐cold PBS to obtain the RhPd‐H@tumor antigen corona complexes. The protein content of the complexes was quantified using the BCA assay to preliminarily characterize the composition of the protein corona.

### 4.10 Cytotoxicity Assay

4T1 and MCF‐7 cells were seeded in 96‐well plates at a density of 1×10^4^ cells per well and cultured for 24 h prior to treatment. The original culture medium was aspirated and replaced with complete medium containing RhPd or RhPd‐H at concentrations of 0, 5, 10, 20, 40, and 60 µg/mL. After 4 h of incubation, the near‐infrared (NIR) treatment group was irradiated with an 808 nm laser (1 W/cm^2^) for 5 min. All groups were then further incubated for 20 h. Subsequently, the supernatant was discarded, and the cells were gently washed once with PBS buffer. A volume of 100 µL of complete medium containing 10% CCK‐8 reagent was added to each well, and the plates were incubated in the dark at 37°C for 1.5 h. Absorbance was finally measured at a wavelength of 450 nm using a microplate reader.

### 4.11 Cellular Uptake Assay

To prepare RB‐labeled RhPd‐H (RhPd‐H@RB), 0.5 mL of RhPd‐H (0.5 mg/mL) was mixed with 1 mg of RB (dissolved in 0.5 mL of ultrapure water). The mixture was shaken at 200 rpm for 24 h at room temperature. Unbound dye was removed by repeated centrifugation and washing until no fluorescent signal was detected in the supernatant. 4T1 and RAW 264.7 cells were cultured to logarithmic growth phase, seeded into 12‐well plates containing glass coverslips at a density of 1×10^5^ cells per well, and incubated overnight. RhPd‐H@RB was then added to each well at a final concentration of 40 µg/mL, followed by another 4 h of incubation. The supernatant was aspirated, and the cells were gently washed once with PBS. An appropriate amount of DiO staining working solution was added, followed by incubation at 37°C for 30 min. The supernatant was discarded, and the cells were gently washed once with PBS. Subsequently, the cells were fixed with 4% paraformaldehyde for 10 min at room temperature, washed again with PBS, and the coverslips were carefully removed. The samples were mounted using an antifade mounting medium containing DAPI, air‐dried in the dark at room temperature, and imaged under a confocal microscope.

### 4.12 Detection of intracellular ROS levels

4T1 cells were seeded in a 96‐well plate at a density of 1×10^4^ cells per well and cultured for 24 h. The old medium was then aspirated, and the cells were divided into six experimental groups: PBS, RhPd, RhPd‐H, PBS + NIR, RhPd + NIR, and RhPd‐H + NIR. Each group was treated with PBS, RhPd (80 µg/mL), or RhPd‐H (80 µg/mL) for 4 h. After incubation, unbound materials were removed by washing with PBS. The NIR‐treated groups were irradiated with an 808 nm laser at a power density of 1 W/cm^2^ for 5 min. Then, 10 µL of culture medium containing DCFH‐DA (DCFH‐DA diluted 1:1000 in RPMI‐1640 blank medium) was added to each well. The plate was gently mixed and incubated at 37°C for 20 min to allow cellular staining. Unbound dye was removed by washing with PBS. Fluorescence intensity (excitation: 488 nm; emission: 525 nm) was measured using a microplate reader at time points from 0 to 240 min.

### 4.13 CLSM Images of 4T1 Cells Using Cell‐Climbing Slices

Briefly, a drop of PBS was placed in each well of a 12‑well plate, followed by a sterile coverslip. Subsequently, 4T1 tumor cells were seeded at a density of 1 × 10^5^ cells per well and cultured in a constant‐temperature incubator. After 24 h, the old medium was removed, and the cells were divided into six groups: PBS, RhPd, RhPd‐H, PBS + NIR, RhPd + NIR, and RhPd‐H + NIR. The corresponding solutions PBS, RhPd (80 µg/mL) or RhPd‐H (80 µg/mL) were added and incubated for 4 h. Unbound materials were then washed away with PBS. Cells in the NIR groups were irradiated with an 808 nm laser at 1 W/cm^2^ for 5 min and returned to the incubator for further treatment.

The following three staining procedures were subsequently performed:

1. After 2 h, 100 µL of medium containing DCFH‐DA (diluted 1:1000 in 1640 medium) and medium containing MitoSOX Red (diluted 1:1000 in 1640 medium) were added to each well. The cells were stained at 37°C for 20 min.
2. 0.5 mL of JC‐1 working solution was added, and staining was performed at 37°C for 20 min.
3. 100 µL of medium containing DHE (diluted 1:1000 in 1640 medium) was added, followed by staining at 37°C for 20 min.

After staining, the supernatant was aspirated, and cells on the coverslips were gently rinsed twice with pre‐cooled sterile PBS. Then, pre‐cooled 4% paraformaldehyde fixative was added to completely cover the coverslips and incubated at room temperature for 15 min. After removing the fixative, the samples were washed three times with PBS. A mounting medium containing DAPI nuclear stain was applied to a glass slide. The coverslip was carefully lifted with sterile forceps, placed cell‐side down onto the center of the slide, avoiding air bubbles. After drying, the samples were observed and imaged under a confocal microscope.

### 4.14 Measurement of Intracellular ATP Levels in Tumor Cells

Intracellular ATP levels were measured in 4T1 cells treated with PBS, RhPd + NIR, or RhPd‐H + NIR. Cells were seeded in 12‐well plates at a density of 1 × 10^5^ cells per well and cultured for 24 h. The medium was then replaced with fresh medium containing PBS, RhPd, or RhPd‐H (80 µg/mL), followed by a further 4 h incubation. Cells in the NIR groups were irradiated with an 808 nm laser (1 W/cm^2^, 5 min) and returned to the incubator. ATP was extracted at 1 and 3 h post‐irradiation. An ATP lysis buffer (volume equal to one‐tenth of the culture medium) was added to each well, and cells were lysed by repeated pipetting. Lysates were centrifuged (12000 × g, 5 min, 4 °C), and the supernatant was collected for analysis. For the ATP assay, 100 µL of ATP detection working solution was added to each well of a 96‐well plate and equilibrated at room temperature for 5 min. Then, 20 µL of sample or ATP standard was added, mixed immediately, and the relative light unit (RLU) was measured after a 2 s delay using a luminometer.

### 4.15 Tissue Distribution of the RhPd‐H in Mice

Female BALB/c mice (6‐8 weeks old) were subcutaneously inoculated with 4T1 cells (2 × 10^6^ cells per mouse) into the left mammary fat pad to establish the tumor model. When the tumor volume reached approximately 100 mm^3^, RB‐labeled RhPd‐H was administered via tail vein injection at a dose of 5 mg/kg. At designated time points post‐injection, the mice were euthanized, and major organs (heart, liver, spleen, lungs, and kidneys) as well as tumor tissues were collected. The ex vivo fluorescence signals of RB in these tissues were visualized and analyzed to evaluate the spatial and temporal distribution of RhPd‐H.

### 4.16 In Vivo Therapeutic Evaluation

Tumor‐bearing mice were first established following the same procedure described in Section 4.14. All animal experiments were approved by Experimental Animal Ethics Committee of Jilin University (Number of permit:KT202406002). When the tumor volume reached approximately 100 mm^3^, the mice were randomly assigned to six groups as follows: (1) PBS, (2) RhPd, (3) RhPd‐H, (4) PBS + NIR, (5) RhPd + NIR, and (6) RhPd‐H + NIR. The corresponding formulations were injected via the tail vein at a dose of 5 mg/kg. At 24 h post‐injection, tumors in the NIR groups were irradiated with an 808 nm laser at a power density of 1 W/cm^2^for 5 min, and a second irradiation was performed after another 48 h. Tumor volumes and body weights were monitored every two days. On day 14, all mice were euthanized, and tumor growth curves were plotted to evaluate therapeutic efficacy.

### 4.17 Biosafety Evaluation

Blood samples were collected from the mice for complete blood panel analysis. The mice were then euthanized, and major organs including the heart, liver, spleen, lungs, and kidneys as well as tumor tissues were harvested. The entire tumor mass was weighed and photographed. All tissue samples were fixed in 4% paraformaldehyde for 24 h, followed by paraffin embedding, sectioning, and H&E staining. Morphological and structural changes in the tissues were examined under an optical microscope to evaluate the safety profile of the treatment regimen.

### 4.18 Preparation of Samples for Flow Cytometry Analysis

The tumor tissue was minced and digested in digestion buffer at 37°C with shaking at 100 rpm for 35 min. The digested tissue was gently pressed through a 70 µm cell strainer on ice, and the resulting cell suspension was resuspended in PBS containing 2% FBS, followed by centrifugation at 450 ×g for 5 min. The pellet was resuspended in 6–10 mL of 35% Percoll separation medium and centrifuged at 400 ×g for 25 min at 22°C with both acceleration and deceleration set to 0. The supernatant was discarded, and the cell pellet was resuspended in PBS with 2% FBS and transferred to a microcentrifuge tube. Then, 600 µL of red blood cell lysis buffer was added for 5 min. The lysis was terminated by adding PBS, and the sample was centrifuged. The pellet was resuspended in 250 µL of PBS containing 2% FBS. A 50 µL aliquot of the cell suspension was incubated with 0.5 µL Fc blocker for 10 min at 4°C. Subsequently, fluorochrome conjugated antibodies were added according to the surface marker staining protocol, and the mixture was incubated at room temperature for 15–20 min protected from light. After staining, cells were washed with PBS containing 2% FBS and centrifuged at 450 ×g for 5 min. The final pellet was resuspended in 200 µL PBS, filtered through a cell strainer, and analyzed by flow cytometry.

Approximately one third of the spleen was weighed and mechanically dissociated through a 70 µm cell strainer to obtain a single cell suspension. The cells were centrifuged at 450 ×g for 5 min and resuspended in 250 µL of PBS with 2% FBS. Two fifths of the cell suspension was treated with Fc blocker for 10 min at 4°C, followed by antibody staining for 40 min. Then, 500 µL of red blood cell lysis buffer was added for 10 min. The reaction was stopped with 1 mL of PBS containing 2% FBS. After centrifugation, the pellet was resuspended in 200 µL of PBS, filtered, and analyzed by flow cytometry.

### 4.19 Statistical Analysis of Data

Graphical representations of data were generated using GraphPad Prism software. Experimental data are expressed as mean ± standard deviation. Significance was determined using a twotailed Student's t‐test. A *p*‐value of less than 0.05 was considered statistically significant. In figures, asterisks indicate significance levels as follows: ^*^ *p* < 0.05, ^**^ *p* < 0.01, ^***^ *p* < 0.001, and ^****^ *p* < 0.0001.

## Author Contributions

Study design, supervision and review: Q.W., M.F.S., J.R.C., D.X.J. and F.F.C. Conducting the experiments: M.F.S., J.R.C., G.Y., Y.W.Y. and H.Y.C. Biological study and data analysis: M.F.S., T.W., and S.J. Chemical synthesis: Y.Y.Z. and Q.W. Drafting of the manuscript: Q.W., M.F.S. and F.F.C. All authors contributed to the work and agreed to the submission of the final manuscript.

## Conflicts of Interest

The authors declare no conflicts of interest.

## Supporting information




**Supporting File**: advs75258‐sup‐0001‐SuppMat.docx.

## Data Availability

The data that support the findings of this study are available from the corresponding author upon reasonable request.
