# Supplementary material for: A Hydrogen‐Releasing Nanozyme Engineers a Mitochondrial ROS Amplifier for Self‐Sustaining Catalytic Immunotherapy
Source: Adv Sci (Weinh). 2026 Apr 10;13(39):e24313. doi: 10.1002/advs.202524313 (PMC13334870; doi:10.1002/advs.202524313)
Supplement: Supplementary file 1 — Supporting File: advs75258‐sup‐0001‐SuppMat.docx. [file ADVS-13-e24313-s001.docx]

**A Hydrogen-Releasing Nanozyme Engineers a Mitochondrial ROS Amplifier for Self-Sustaining Catalytic Immunotherapy**

Mingfan Shi^1#^, Jingrui Cao^2#^, Tong Wu^5^, Guang Yang^6^, YaWen Yang^6^, Shixin Zhang^1^, Wenwen Su^1^, Hongyu Chu^3^, Yangyang Zhao^1^, Shan Jiang^1^, Qiong Wu^1*^, Dongxu Jiao^4*^, Fangfang Chen^1*^

*^1^ Key Laboratory of Pathobiology, Ministry of Education, Nanomedicine and Translational Research Center, China-Japan Union Hospital of Jilin University, 126 Sendai Street, Changchun 130033, Jilin, China*

*^2^ Huzhou Central Hospital, The Fifth School of Clinical Medicine of Zhejiang Chinese Medical University, 1558 Sanhuan North Road, Wuxing District, Huzhou, 313000, Zhejiang Province, China*

*^3^ Department of Gastrointestinal, Colorectal and Anal Surgery, China-Japan Union Hospital of Jilin University, 126 Sendai Street, Changchun 130033, Jilin, China*

*^4^ College of Chemistry, Chemical Engineering and Resource Utilization, Northeast Forestry University, Harbin 150040, China*

*^5^ The First Research Laboratory, Changchun Institute of Biological Products Co.,Ltd., 1607 Chaoran Street, Changchun 130103, Jilin, China*

*^6^ JILIN Cancer Hospital, 1018, Huguang Road, Changchun 130012, Jilin, China*

# These authors contributed equally: Mingfan Shi, Jingrui Cao

Corresponding author email: [qiong_wu@jlu.edu.cn,](mailto:qiong_wu@jlu.edu.cn,) [dxjiao_0518@163.com,](mailto:dxjiao_0518@163.com,) cff@jlu.edu.cn

Figure S1. Zeta potential of RhPd and RhPd-H.


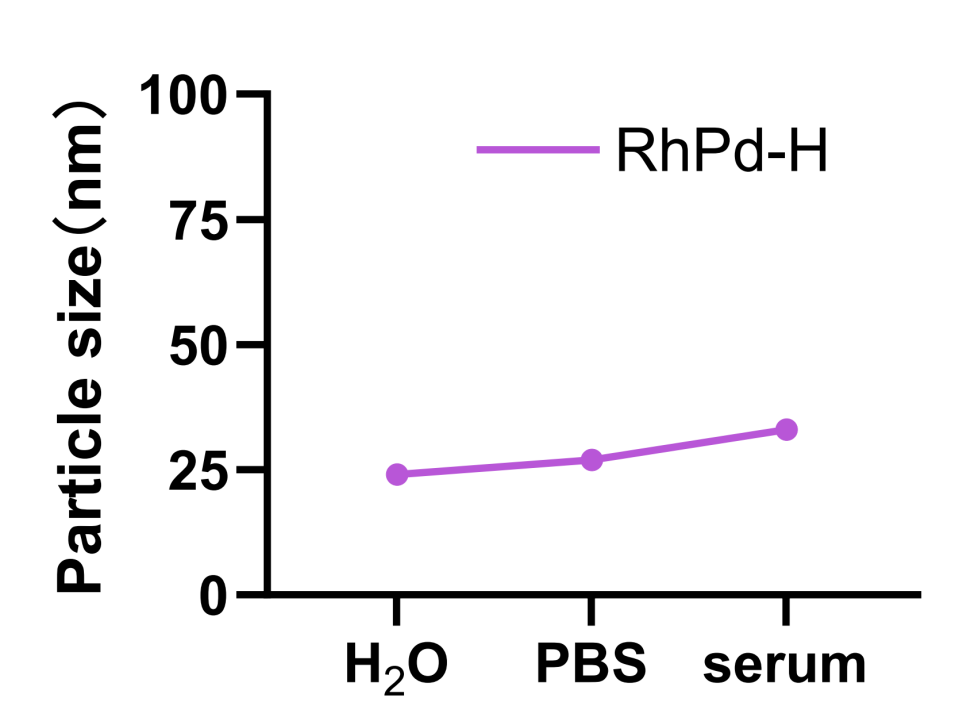


Figure S2. Nanoparticle tracking analysis (NTA) of RhPd-H nanoparticles in different solutions.


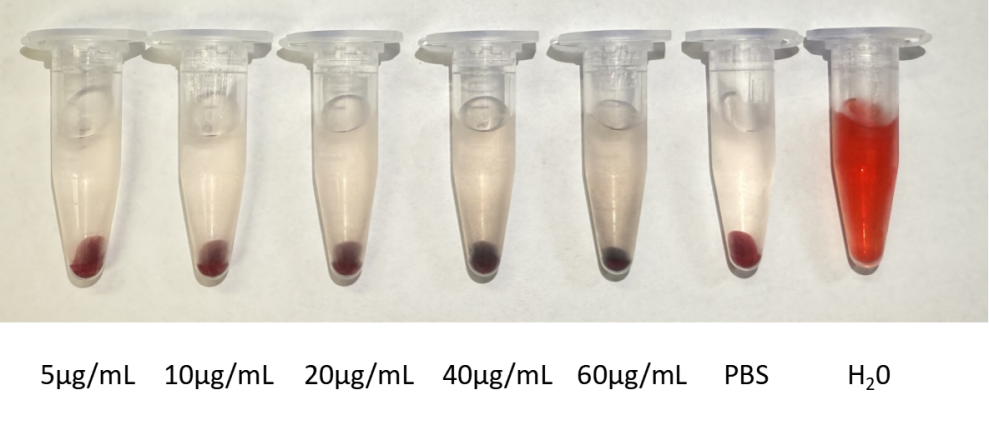


Figure S3. Photograph and quantitative analysis of hemolysis assays for RhPd-H (n=4).

Figure S4. Comparison of photothermal conversion capabilities between RhPd and RhPd-H.


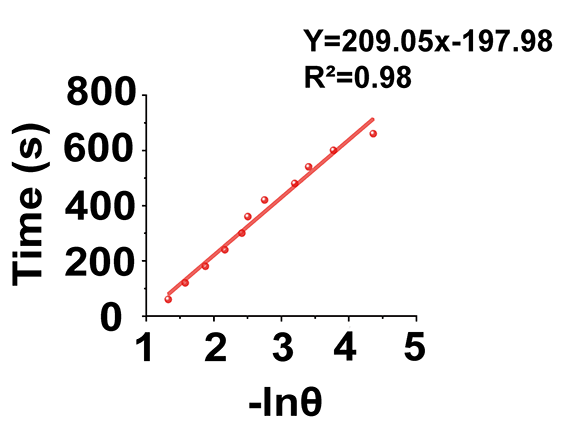


Figure S5. Photothermal conversion ability of RhPd-H. The cooling period plot against the negative natural logarithm of the temperature according to the cooling stage.


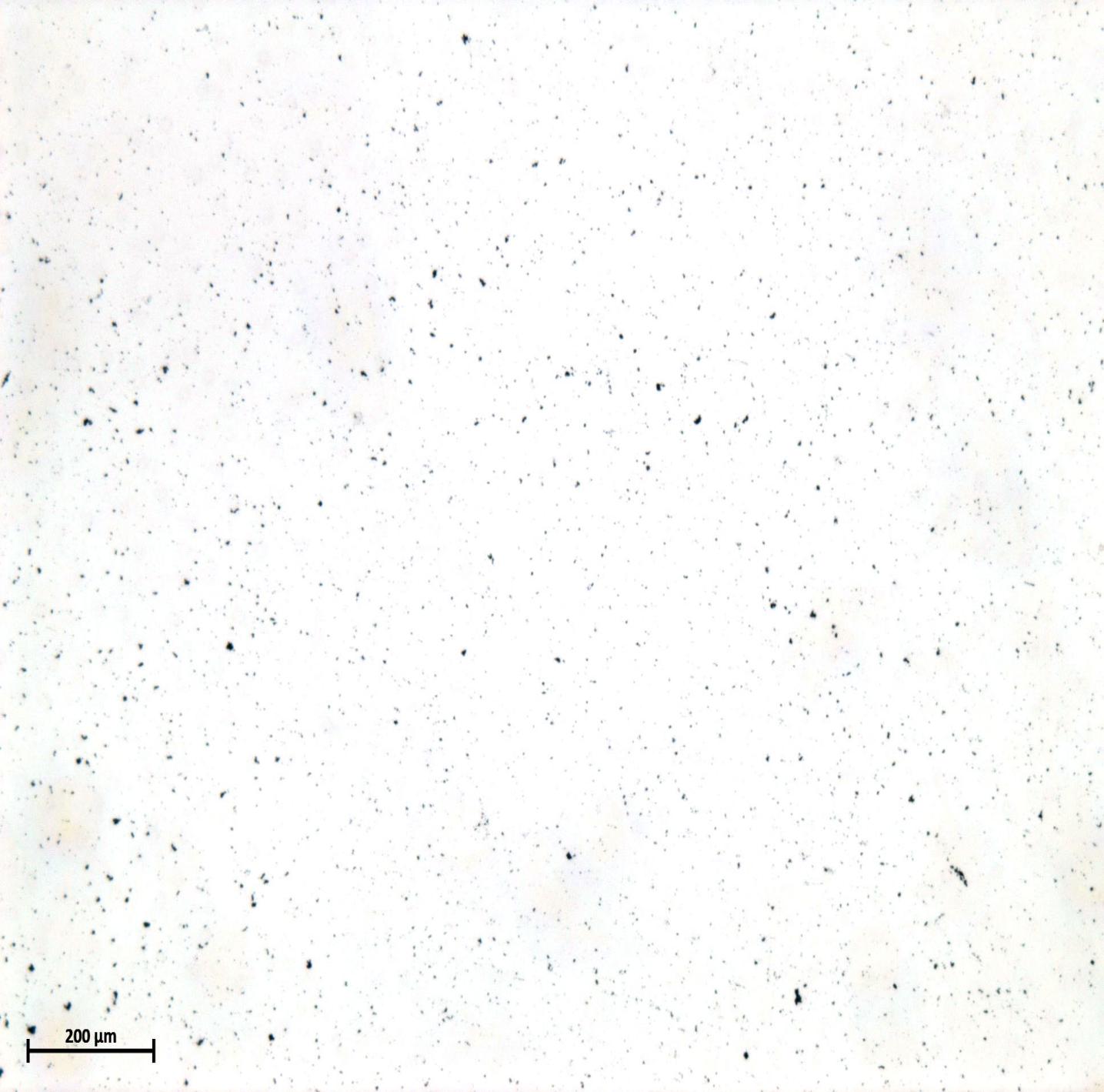


RhPd

RhPd-H


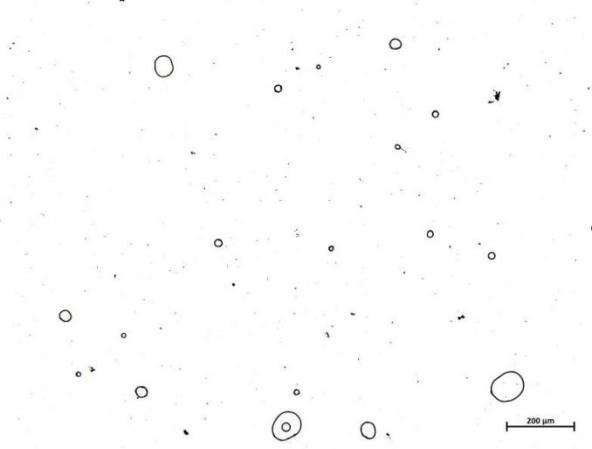


200 μm

Figure S6. Optical microscopy image of hydrogen bubbles released from RhPd-H and RhPd under NIR irradiation. Scale bar: 200 μm.


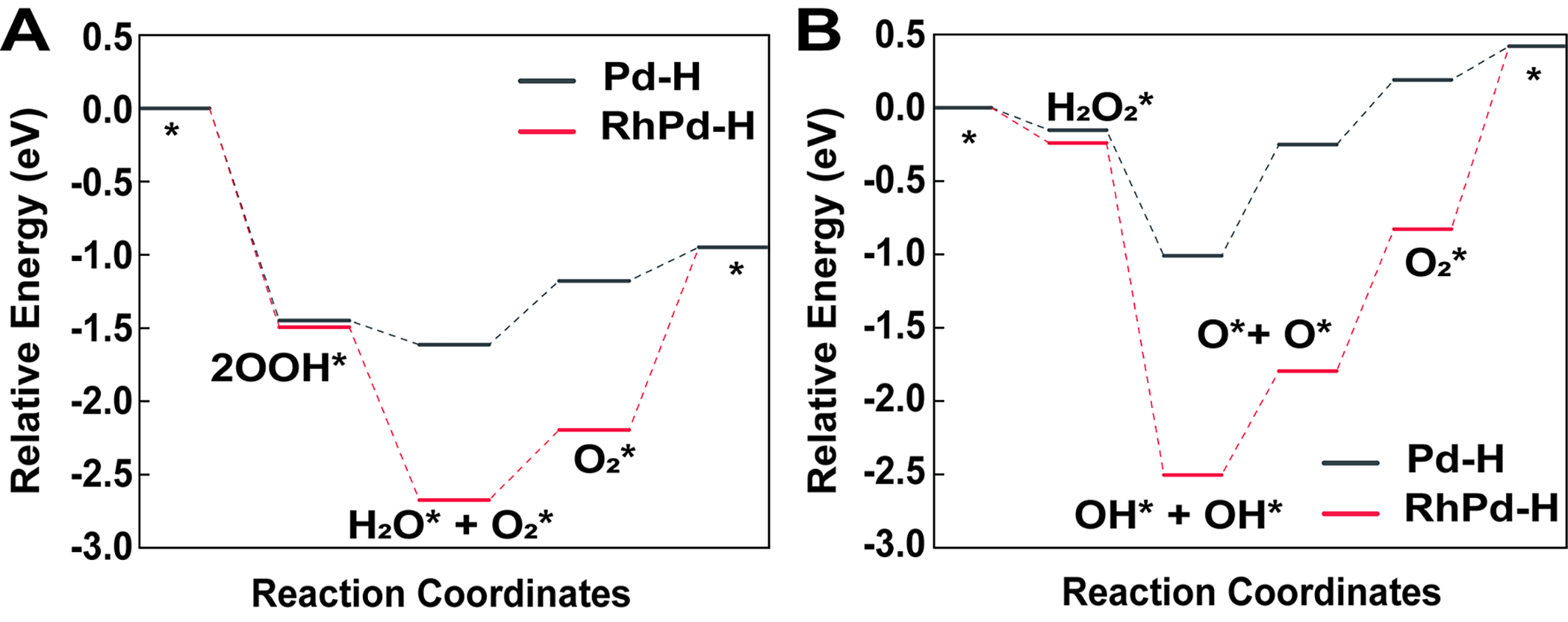


Figure S7. Corresponding free energy diagrams of Pd-H and RhPd-H NPs for SOD- (A) and CAT-(B) like reaction.


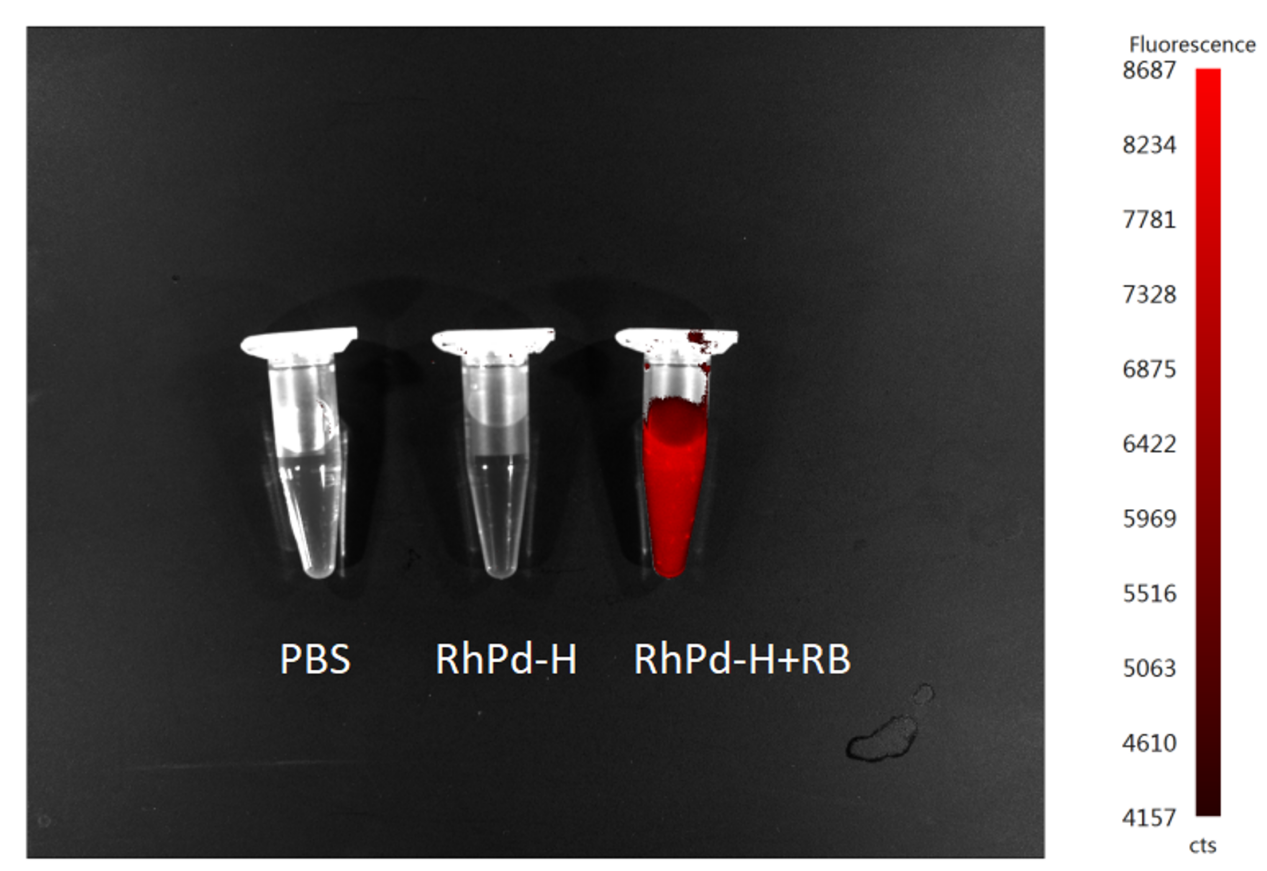


Figure S8. Fluorescence images of PBS, RhPd-H, and RhPd-H labeled with RB.


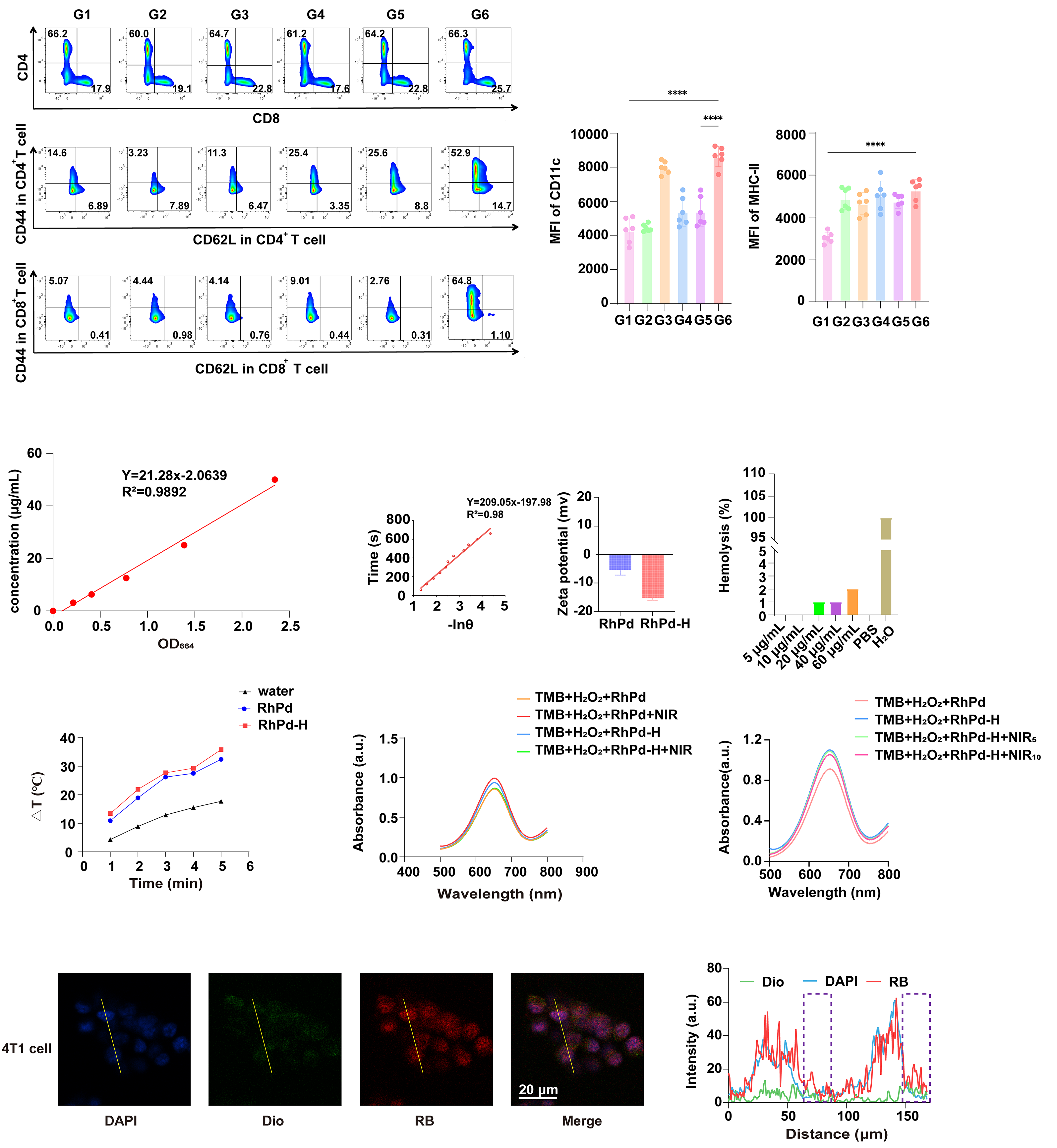


Figure S9. Comparison of POD-like activity of RhPd and RhPd-H pretreated with NIR irradiation (5 min or 10 min).


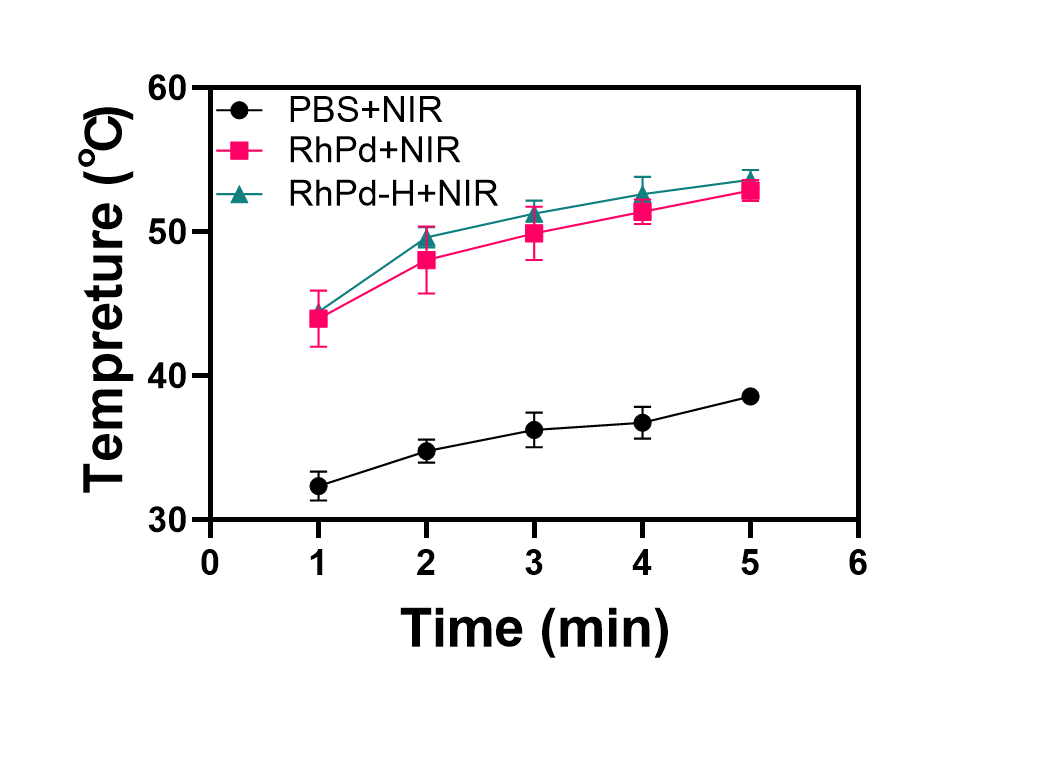


Figure S10. Thermal imaging data during treatment.


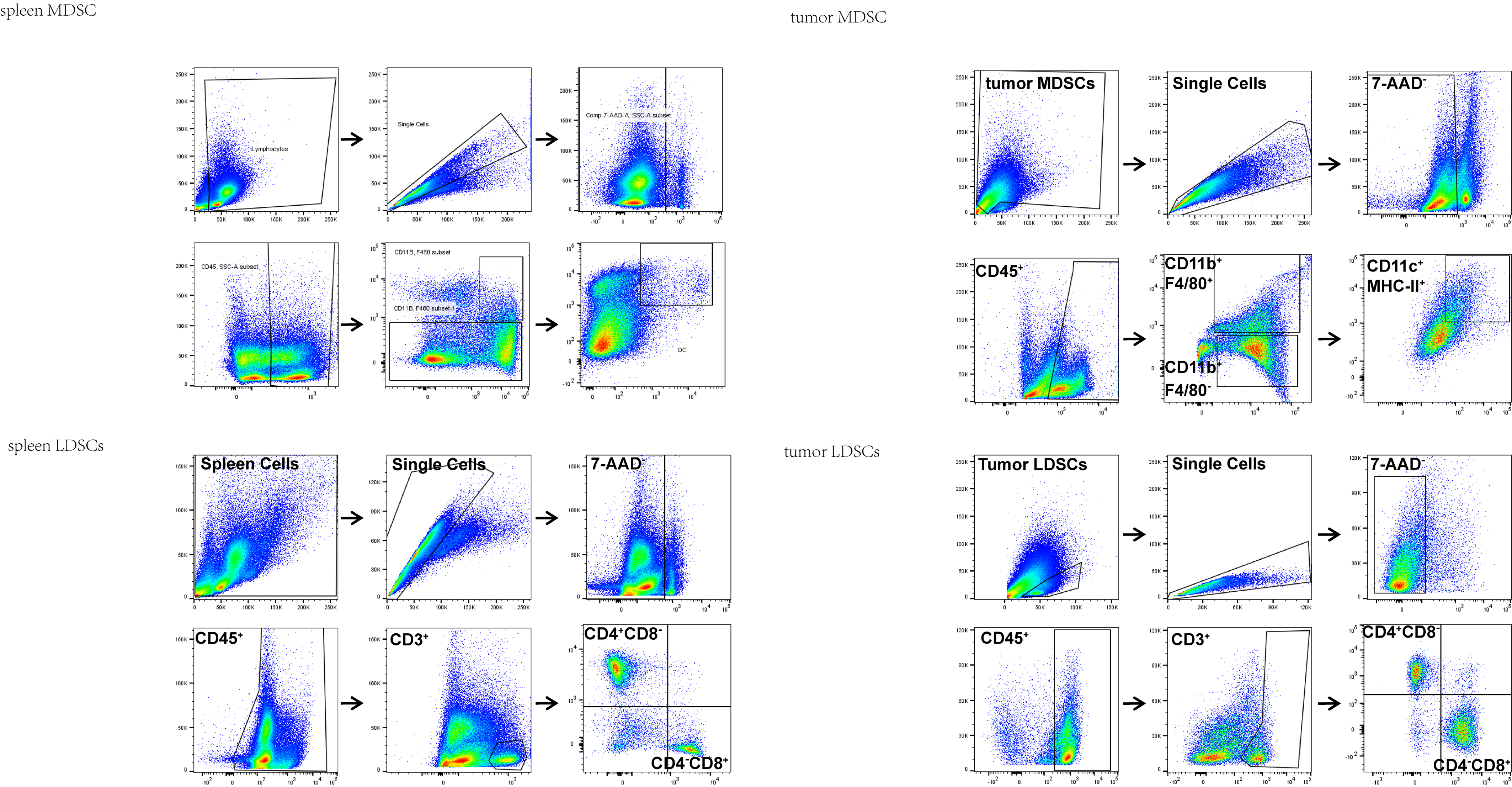


Figure S11. Gating strategy for MDSC analysis in tumors.


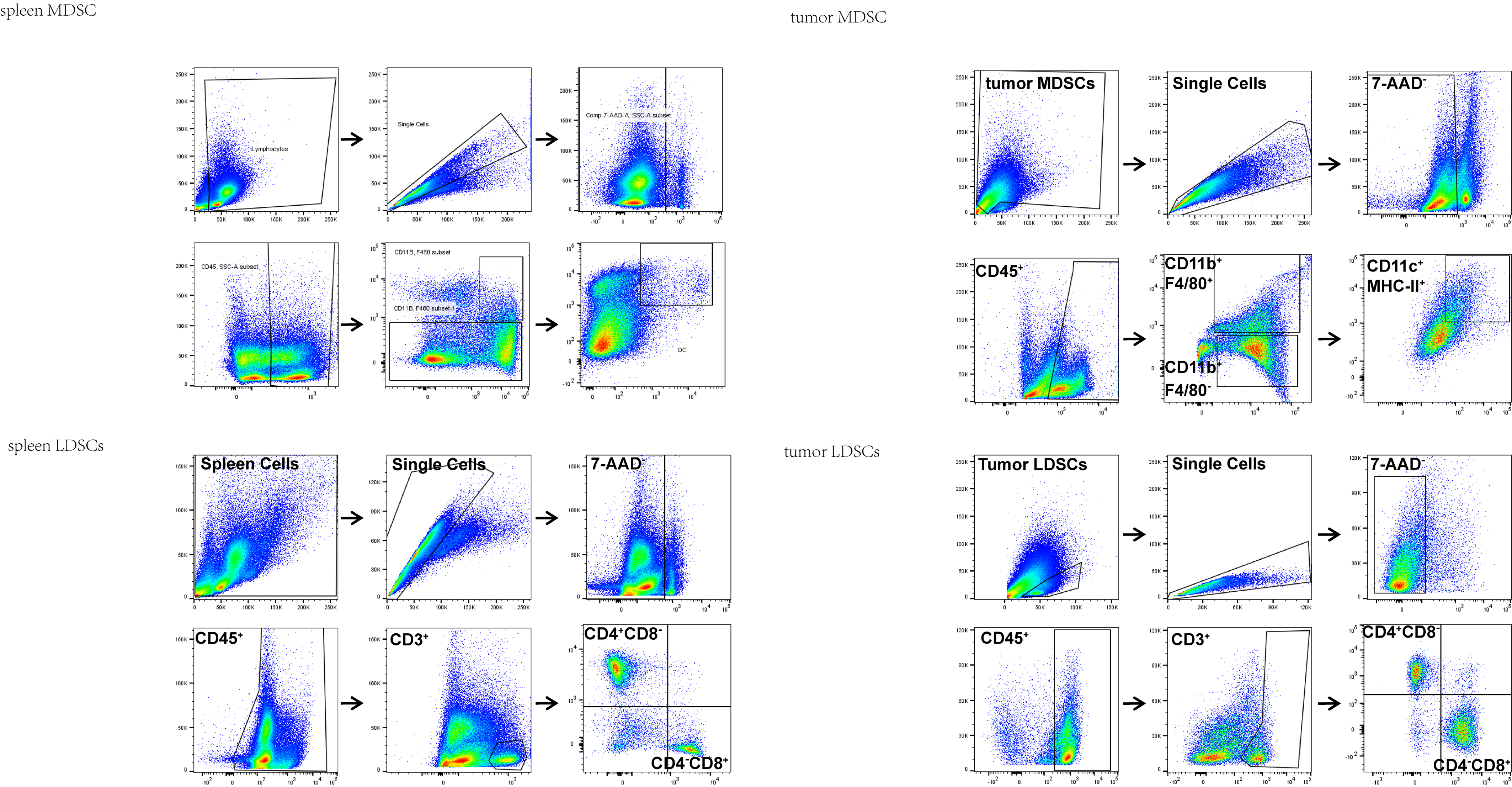


Figure S12. Gating strategy for LDSCs analysis in tumors.


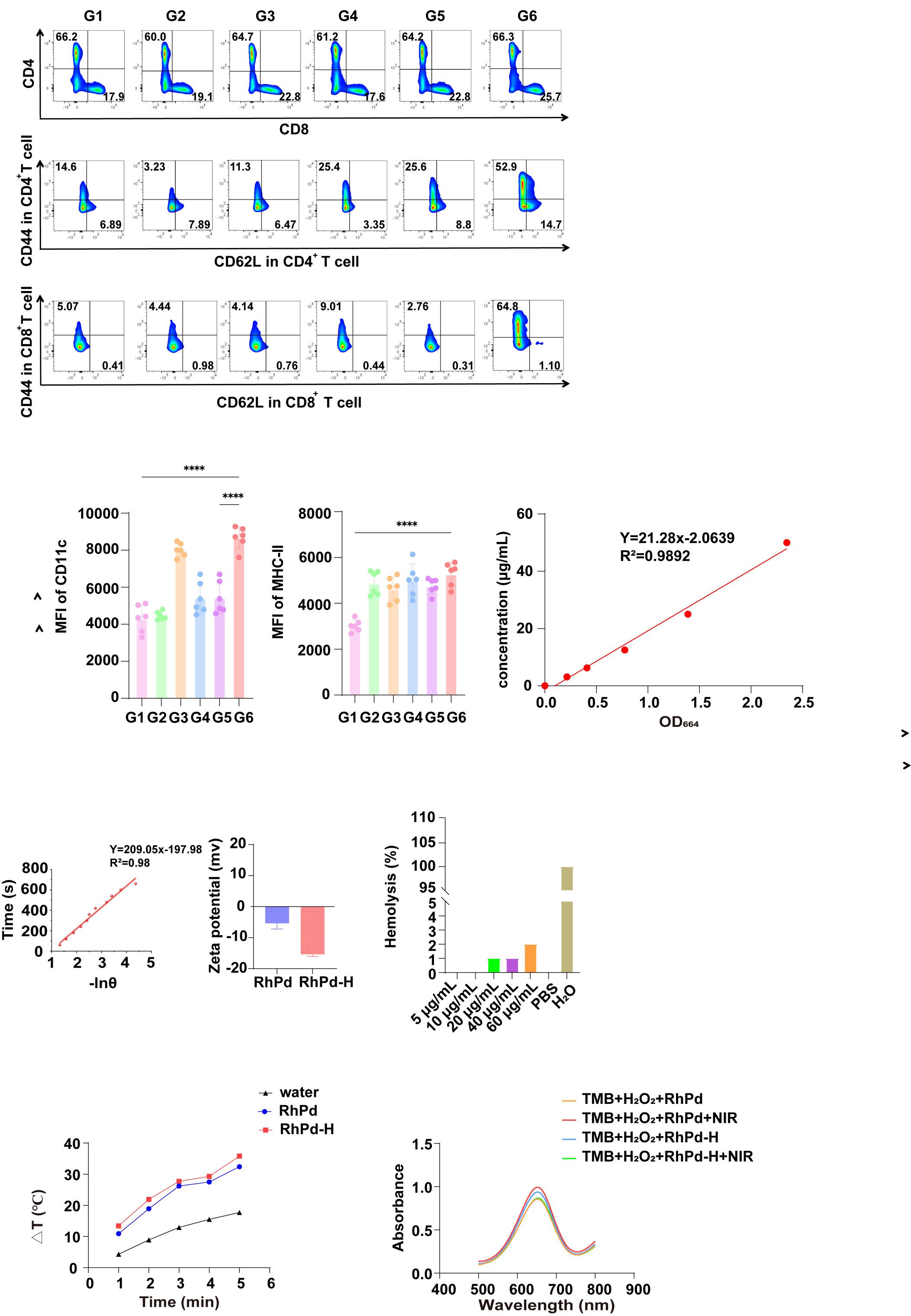


Figure S13. Flow cytometry result plots of immune cells in mouse spleen (n=6). Flow cytometry analysis of tumor-infiltrating CD4^+^ and CD8^+^ T cells from different treatment groups. Treatment groups: G1, PBS; G2, RhPd; G3, RhPd-H; G4, PBS+NIR; G5, RhPd+NIR; G6, RhPd-H+NIR.


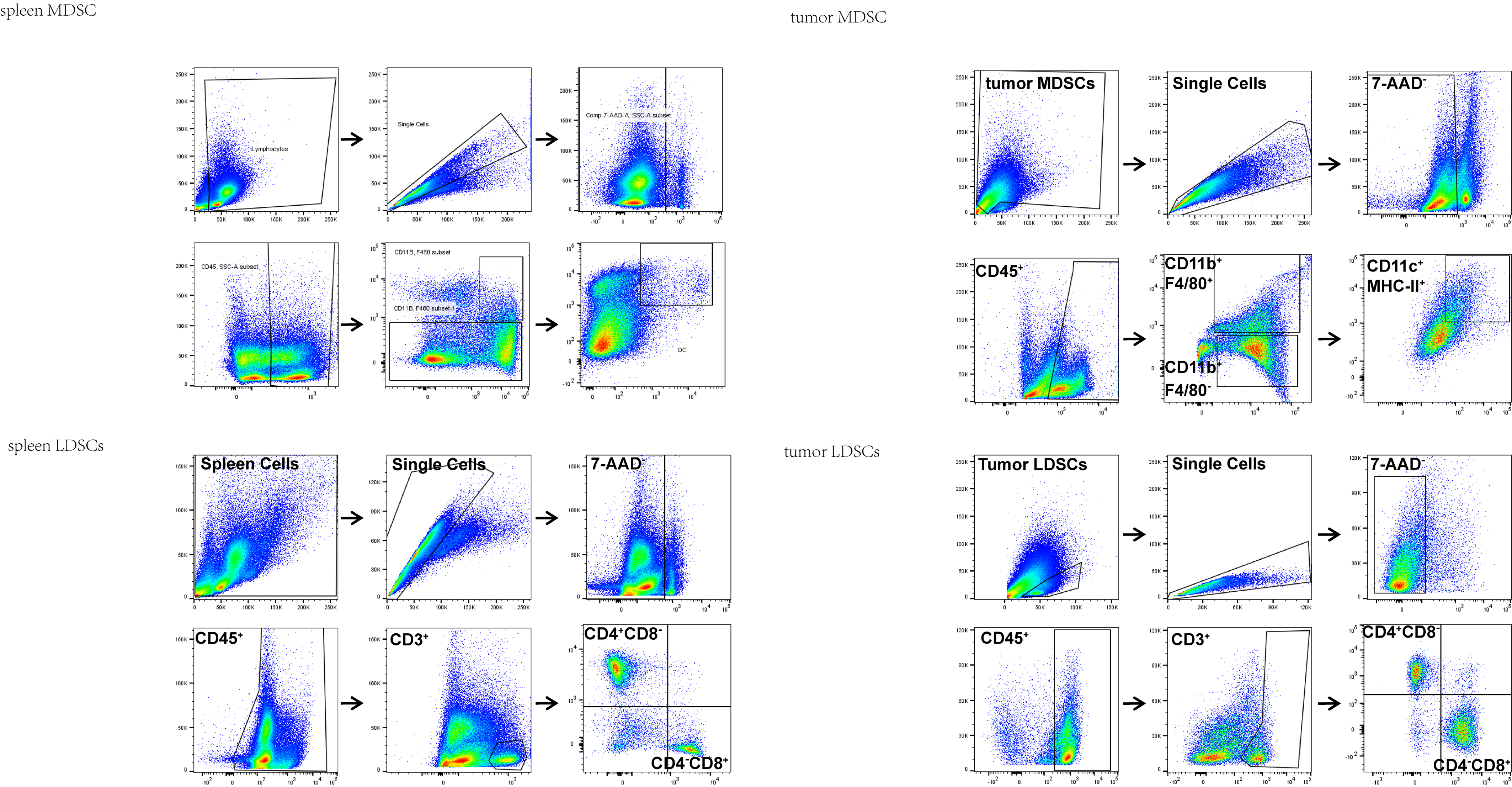


Figure S14. Gating strategy for LDSCs analysis in spleen.
